# The plant hormone abscisic acid regulates the growth and metabolism of endophytic fungus *Aspergillus nidulans*

**DOI:** 10.1038/s41598-018-24770-9

**Published:** 2018-04-25

**Authors:** Gangming Xu, Suiqun Yang, Linghong Meng, Bin-Gui Wang

**Affiliations:** 1 Key Laboratory of Experimental Marine Biology, Institute of Oceanology, Chinese Academy of Sciences, Nanhai Road 7, Qingdao, 266071 People’s Republic of China; 2 Laboratory of Marine Biology and Biotechnology, Qingdao National Laboratory for Marine Science and Technology, Nanhai Road 7, Qingdao, 266071 People’s Republic of China; 3 University of Chinese Academy of Sciences, 19A Yuquan Road, Beijing, 100049 P. R. China

## Abstract

Plant hormones are well known chemical signals that regulate plant growth, development, and adaptation. However, after comparative transcriptome and metabolite analysis, we found that the plant hormone abscisic acid (ABA) also affect the growth and metabolism of endophytic fungus *Aspergillus nidulans*. There were 3148 up-regulated and 3160 down-regulated genes identified during 100 nM ABA induction. These differentially expressed genes (DEGs) were mainly involved in: RNA polymerase and basal transcription factors; ribosome biogenesis, protein processing, proteasome, and ubiquitin mediated proteolysis; nucleotide metabolism and tri-carboxylic acid (TCA) cycle; cell cycle and biosynthesis of secondary metabolites. Production of mycotoxins, which have insect-resistance or anti-pathogen activity, was also changed with ABA induction. This study provides the first global view of ABA induced transcription and metabolite changes in endophytic fungus, which might suggest a potential fungus-plant cross-talk via ABA.

## Introduction

During long-term of co-evolution, a closely mutualistic relationship was developed between the endophytic fungi and their host plant^1,2^. Plant hormones (phytohormones) are signal molecules produced in plant with low concentration that regulate plant growth, development, and adaptation^3,4^. Some phytohormones, such as gibberellins (GAs), indoleacetic acid (IAA) and strigolactones (SLs), are also biosynthesized in microbes^5–7^. GA was first isolated from the rice fungal pathogen *Gibberella fujikuroi*^7^. IAA induced invasive growth of *Saccharomyces cerevisiae*^8^. Strigolactone induced hyphal branching of *Gigaspora margarita*, which is an arbuscular mycorrhizal fungus^6^. However, effects of phytohormones on the growth and metabolism of endophytic fungi, and their signaling roles during plant-microbe interactions are rarely reported^5,9^.

The phytohormone abscisic acid (ABA) is a plant signaling molecule mediating seed dormancy, bud growth, and adaptation to environmental stresses^10,11^. ABA is biosynthesized in plants, and also in some pathogen fungi, such as *Botrytis cinerea*^12–14^. The ABA biosynthesis is via the 2-C-methyl-D-erythritol-4-phosphate (MEP) pathway in plants, whereas mainly by the mevalonic acid (MVA) pathway in fungi^10,12^. Phytopathogens may produce ABA to suppress the plant immune responses^15,16^. It’s suggested that ABA may act as signaling molecule during inter-species communication^15^. However, whether the host plant ABA would affect the growth and adaptation of endophytic fungi is still elusive.

During our earlier screening by small molecules (fungal signals, antibiotics, and phytohormones), we found that plant hormones affected the production of secondary metabolites in endophytic fungi. In this study, we performed RNA-seq analysis of the endophytic fungus *Aspergillus nidulans* MA-143 induced by ABA. The gene expression profiling of 30,242 total unigenes were annotated in 7 databases. After comparative transcriptomic analysis, about 6308 differentially expressed genes (DEGs) were identified during ABA induction. This study presents the first transcriptome changes of ABA effects on endophytic fungus, which might give some new idea about the ecological function of plant hormones during plant-microbe interactions.

## Results

### ABA affects the growth and secondary metabolites of *A. nidulans*

**Figure 1:**
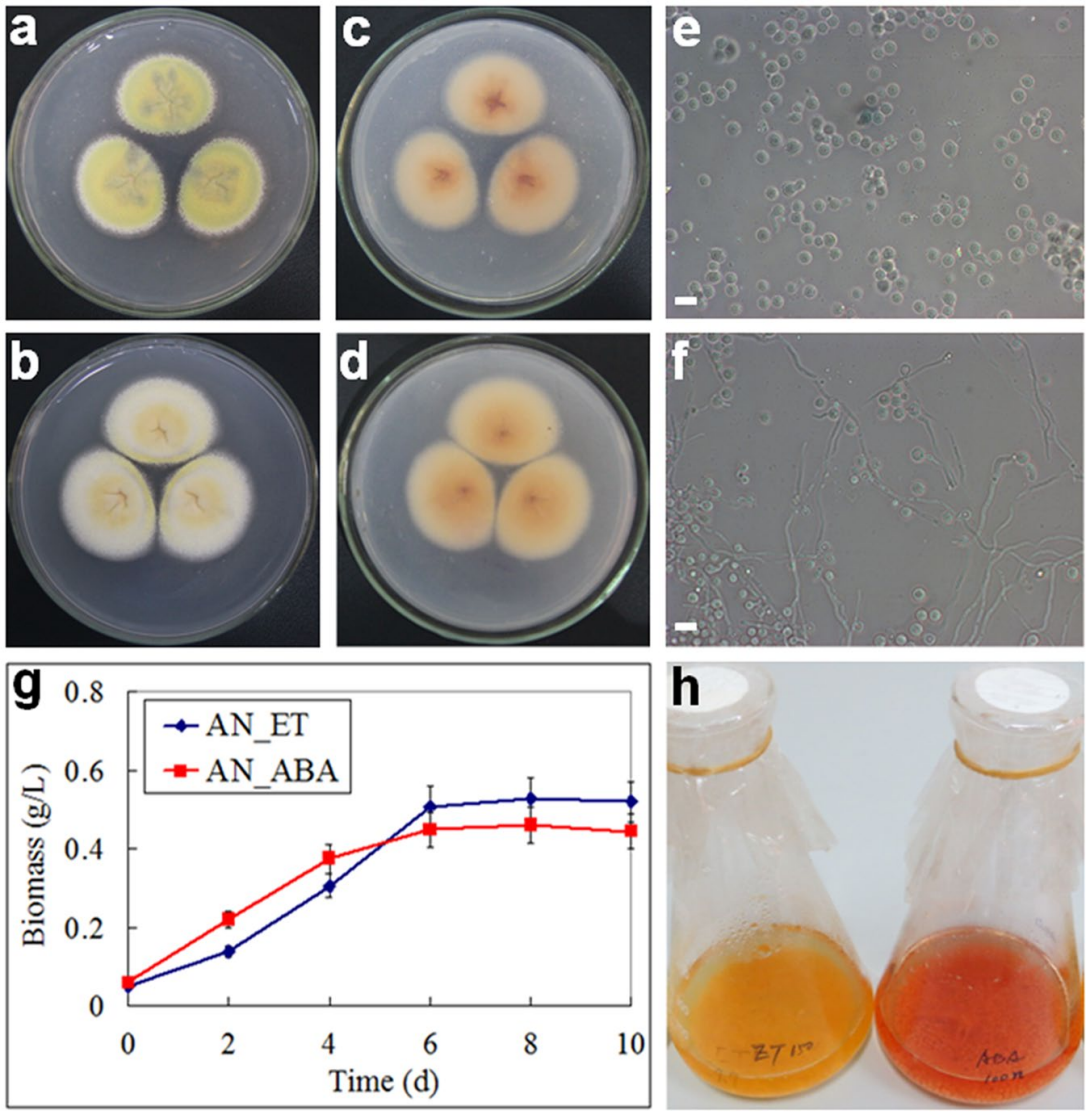
Phenotypic differences of *A. nidulans* with and without ABA induction. (**a**) *A. nidulans* with 0.1% ethanol (AN_ET) on ICI plate 10d front; (**b**) *A. nidulans* with 100 nM ABA (AN_ABA) on ICI plate 10d front; (**c**) AN_ET on ICI plate 10d back; (**d**) AN_ABA on ICI plate 10d back; (**e**) AN_ET in ICI liquid culture 1d; (**f**) AN_ABA in ICI liquid culture 1d; Bars 10 μm. (**g**) Growth of *A. nidulans* with and without ABA induction; (**h**) Fermentation of *A. nidulans* with and without ABA induction in flasks for 10d.

**Figure 2:**
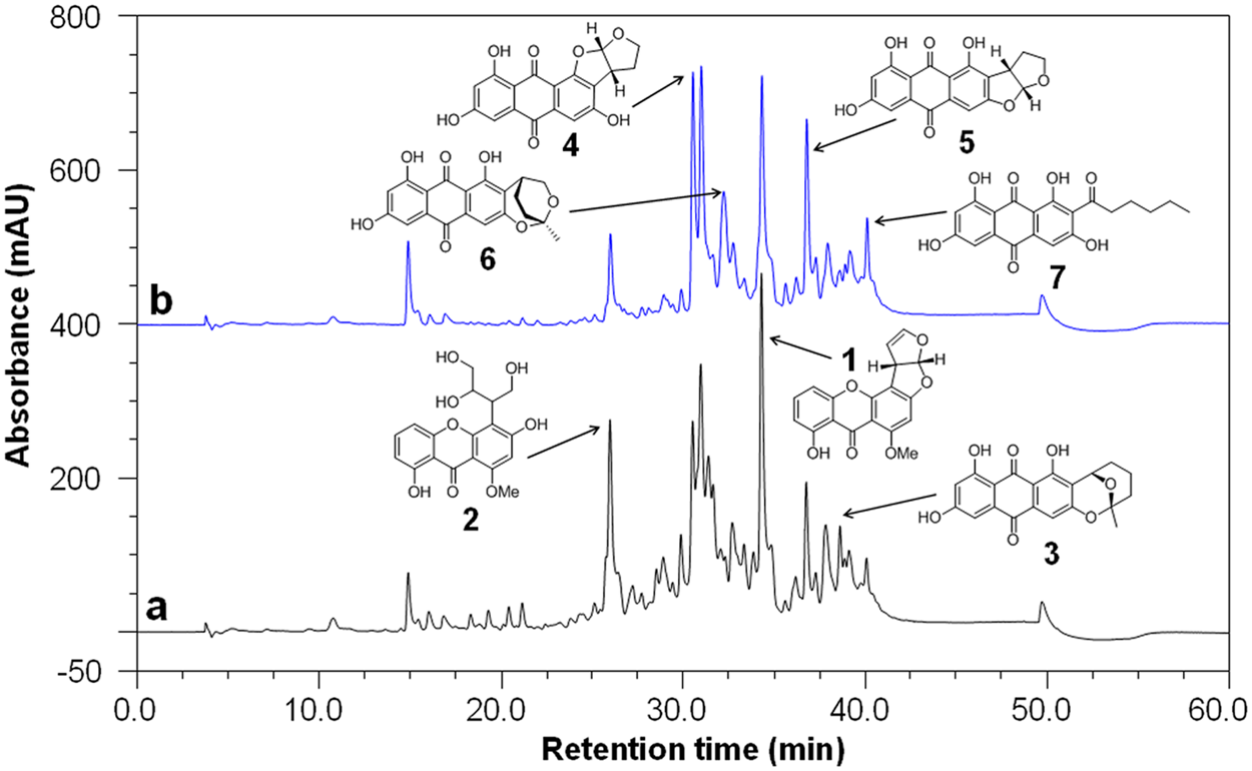
Metabolite profiles of *A. nidulans* with and without ABA induction. (**a**) *A. nidulans* with ethanol (AN_ET); (**b**) *A. nidulans* with ABA (AN_ABA). Compounds isolated and identified: **1** sterigmatocystin, **2** secosterigmatocystin, **3** averufin, **4** new compound, **5** vericolorin B, **6** paeciloquinone E, **7** norsolorinic acid.

The phenotypic differences of *A. nidulans* with and without ABA induction are shown in Fig. 1. On solid medium plates, the fungal growth and spore formation with 100 nM ABA induction were slightly different from the controls (Fig. 1a–d). In liquid cultures, ABA at 100 nM might promote the spore germination of *A. nidulans* (Fig. 1e,f). Growth curves of *A. nidulans* in liquid cultures indicated that ABA at 100 nM promoted fungal growth at first, while reduced the biomass accumulation of fungal mycelium in the end (Fig. 1g). The pigments of mycelium on solid plates and in liquid fermentations of *A. nidulans* with ABA induction were also slightly different (Fig. 1c,d,h). HPLC analysis of crude extracts of liquid cultures indicated that some peaks have changed with ABA induction (Fig. 2). By searching our natural products database, production of anthraquinones and alkaloids were changed with ABA induction^17,18^. These phenotypic differences suggest that ABA might affect the growth and secondary metabolites of endophytic fungus *A. nidulans*.

### Transcriptome sequence, gene functional annotation and classification

Transcriptome sequencing of *A. nidulans* and identification of DEGs with ABA were done following standard pipelines^19,20^. The quality control summary of RNA-seq clean data are shown in Supplementary Table S1. Pearson correlations between samples are shown in Fig. S1. Gene functional annotations were done in 7 databases: NR (NCBI non-redundant protein sequences), NT (NCBI nucleotide sequences), Pfam (Protein family), KOG (euKaryotic Ortholog Groups), GO (Gene Ontology), Swiss-prot, and KEGG (Kyoto Encyclopedia of Genes and Genomes)^21–24^. As shown in Fig. S2, about 30,242 total unigenes were successfully annotated. Species classification in the NR database (Fig. S3) indicated that the *A. nidulans* strain MA-143 was most closely to the model organism *A. nidulans* FGSC A4^18,25^. The principle biological functions of these genes were mapped to the GO terms of cellular process, metabolic process, cell part, organelle, binding and catalytic activity (Fig. S4). In the KOG function classification, these annotated genes were grouped mainly to posttranslational modification, protein turnover, chaperones; translation, ribosomal structure and biogenesis; energy production and conversion; signal transduction mechanisms; amino acid transport and metabolism etc (Fig. S5). In the KEGG classification, these annotated genes were mainly mapped to translation, carbohydrate metabolism, amino acid metabolism, signal transduction, energy metabolism, lipid metabolism, folding-sorting and degradation, transport and catabolism, cell growth and death, endocrine system etc (Fig. S6).

### Identification and enrichment analysis of DEGs in *A. nidulans* induced by ABA

**Figure 3:**
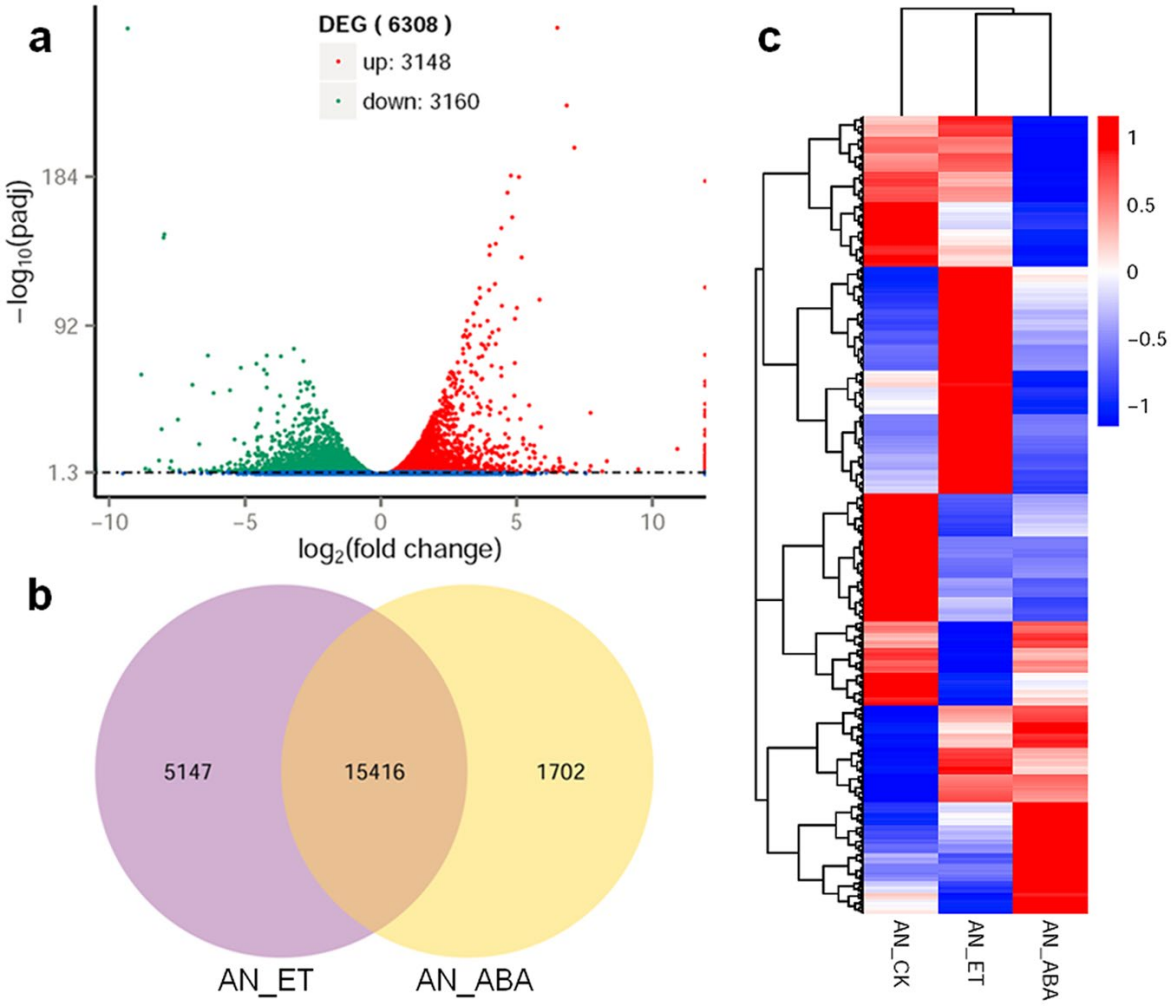
ABA induced DEGs analysis. (**a**) DEGs filter of AN_ABA vs AN_ET by volcano plot; (**b**) Co-expression analysis of AN_ABA vs AN_ET by Venn diagram; (**c**) Heat map cluster analysis of DEGs; *A. nidulans* (AN_CK), *A. nidulans* with ethanol (AN_ET), and *A. nidulans* with ABA (AN_ABA).

**Figure 4:**
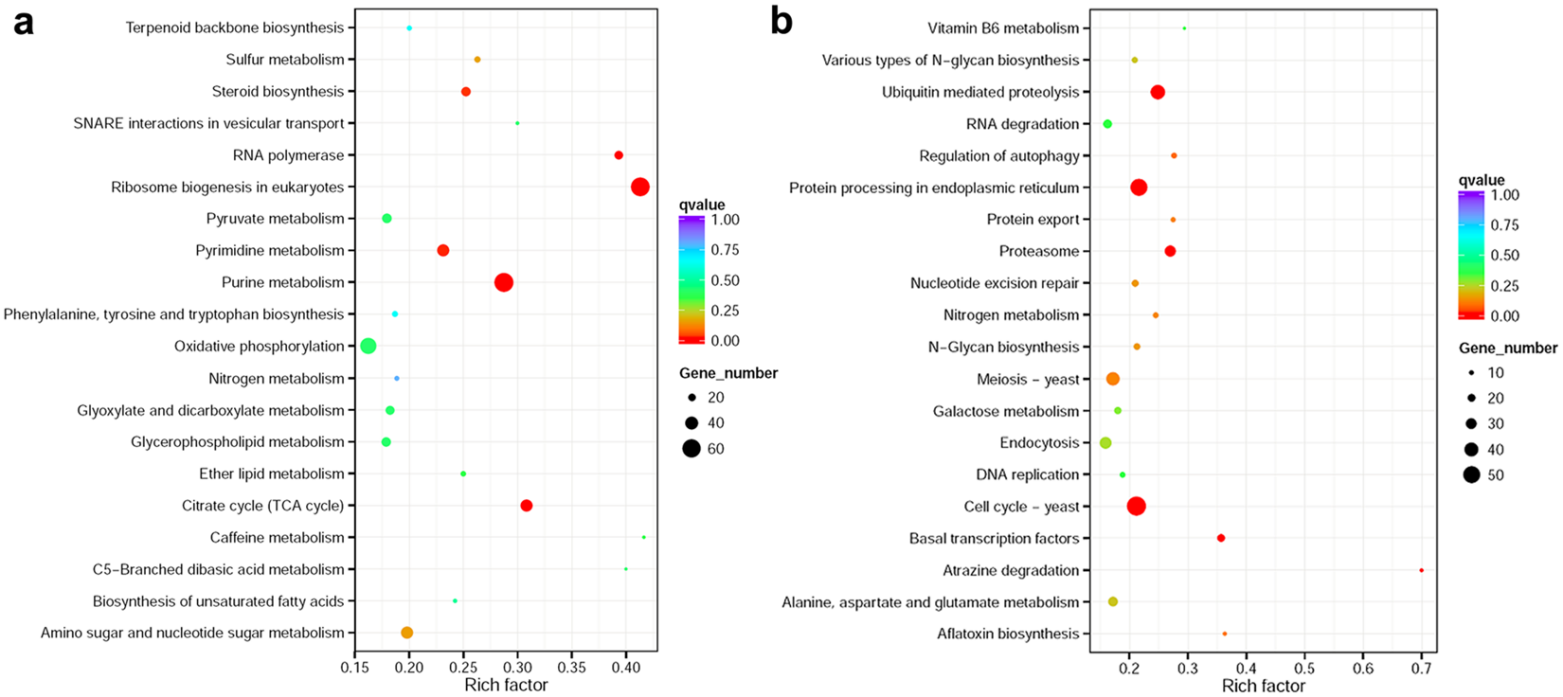
KEGG enrichment of DEGs by ABA induction. DEG (AN_ABA vs AN_ET) enriched KEGG pathways were compared by scatter plot. (**a**) up-regulated; (**b**) down-regulated.

About 6308 out of all the 30,242 annotated unigenes were differentially expressed in *A. nidulans* induced by ABA. Transcription levels of 3148 up-regulated and 3160 down-regulated genes were identified in *A. nidulans* with ABA (Fig. 3a). There were 1702 annotated genes expressed only by ABA induction (Fig. 3b). Heat map clustering of these DEGs indicated that there were significant differences after induced by ABA (Fig. 3c). These ABA induced DEGs were further enriched by GO and KEGG enrichment analysis. The enriched GO terms of these DEGs mainly include: small molecule metabolic process, organonitrogen compound metabolic process, cellular metabolic process, cell part, intracellular part, and transferase activity etc (Fig. S7). The up-regulated KEGG pathways mainly include: ribosome biogenesis in eukaryotes, purine metabolism, TCA cycle, RNA polymerase, pyrimidine metabolism, and steroid biosynthesis (Fig. 4a). Whereas, the down-regulated pathways mainly include ubiquitin mediated proteolysis, cell cycle, basal transcription factors, protein processing in endoplasmic reticulum, and proteasome (Fig. 4b). The most remarkable transcriptional changes induced by ABA are described in details below.

### DEGs encoding RNA polymerases and basal transcription factors

The eukaryotic RNA polymerases (Pol I-III) are the central apparatus that synthesize rRNA, mRNA and tRNA^26,27^. About 26 out of 61 gene homologs of eukaryotic Pol I, II and III subunits were increased significantly with ABA (Table S2), including Pol I, II and III common subunits (ABC2 and ABC4), Pol I core subunits (A1, A2, AC1 and AC2), Pol I specific subunits (A43 and A49), Pol III core subunits (C1, C2, AC1 and AC2), Pol III specific subunits (C3, C11 and C25), and Pol II core subunits (B1 and B3). While none of the Pol II specific subunits (B4, B7 and B9) have changed. These indicated that the gene transcription profiles in *A. nidulans* might be increased with ABA induction.

Basal transcriptional factors (BTF), together with Pol and mediators, constitute the transcriptional apparatus to activate and regulate gene transcriptions^28,29^. There were 25 out of 56 BTF gene homologs in the DEGs induced by ABA (Table S3). Only 5 gene homologs (*TBP, TAF14, XPB, CDK7* and *CCNH*) were up-regulated; whereas 20 BTF gene homologs for Pol II (TFIIA, TFIID, TFIIE, TFIIF and TFIIH) were repressed. These might lead to a pleiotropic gene regulation in the fungal cell responding to environmental signals and stresses.

### DEGs involved in ribosome biogenesis, protein processing, proteasome, and ubiquitin mediated proteolysis

Eukaryotic ribosome biogenesis involves the production and assembly of rRNAs and ribosomal proteins, which makes the cellular factories for protein synthesis^30,31^. Up to 74 of all the 150 genes for ribosome biogenesis (40S and 60S subunits) in eukaryotes were extensively changed in *A. nidulans* with ABA (Table S2). There were 62 up-regulated gene homologs, which involved in rRNA modification (*NOP1, NOP58, DKC1, NHP2, GAR1*, and *Nop10*), 90S pre-ribosome (*UTP5, UTP4, UTP10, UTP15, NAN1, UTP6, PWP2, Dip2*, and *MPP10*), cleavages (*UTP24, UTP14, Rnt1, EMG1, Bms1, Rcl1*, *KRE33*, Nog1, Nug1/2, and NOP4), and export (*Nob1, HRR25, Ran, Tap, NMD3, Rio2, LSG1*, and *Drg1*) etc. These changes suggested that related protein syntheses in *A. nidulans* might be increased with ABA induction.

Protein processing in endoplasmic reticulum involves package and transport correctly folded proteins and degradation of misfolded proteins with the help of chaperones^32^. There were 70 out of 231 gene homologs in protein processing changed by ABA induction (Table S3). About 20 gene homologs were up-regulated, such as protein transport protein Sec. 13, Derlin-1, and molecular chaperone HSP90 etc; whereas 50 gene homologs were down-regulated, including protein transport protein Sec. 61, oligosaccharyl-transferase complex (OSTs), mannosyl-oligosaccharide glucosidase (Glc1), heat shock protein (Hsp40 and Bip), protein disulfide-isomerase (PDIs), nuclear protein localization protein 4 (Npl4), and endoribonuclease IRE1 etc. These suggested that ribosome anchor (OSTs and Glc1), protein recognition by luminal chaperones (Hsp40 and Bip), and protein targeting degradation (PDIs and Npl4) might be repressed.

Proteasome, which has a 20S proteolytic core and ancillary factors, is a protein- degrading apparatus involved in various cellular functions^33^. There were 36 out of 115 gene homologs in proteasome changed by ABA induction (Table S3). Only 5 gene homologs were up-regulated, such as coding genes for 20S proteasome subunit α1 and proteasome activator subunit 4 (PA200) etc; whereas 31 gene homologs were down-regulated, including genes for 20S proteasome core subunits (α2-7, β1-4 and β7), regulatory particles PA700 (Rpt1-5, Rpn1-2, Rpn5-6, Rpn8 and Rpn12), and proteasome maturation protein POMP etc. These suggested that the proteasome might be repressed with ABA induction.

Ubiquitin mediated proteolysis regulates the breakdown of intracellular proteins by proteasome with extreme specificity, which involved in numerous cellular processes like cell cycle, DNA repair, and protein quality control etc^34^. There were 57 out of 169 gene homologs in the ubiquitin mediated proteolysis signaling pathway altered by ABA induction (Table S3). Only 15 gene homologs encoding ubiquitin-conjugating enzyme E2 (*UBE2I* and *UBE2J2*) and ubiquitin ligase E3 (*ARF-BP1, UBE4B, PRP19, CHIP* and *Apc1* etc) were up-regulated; whereas 42 gene homologs encoding ubiquitin-activating enzyme E1 (*UBLE1A* and *UBLE1B*), E2 (*UBE2A, UBE2D_E, UBE2G1, UBE2M* and *UBE2R*), and E3 (*UBE3C, TRIP12, NEDD4, Apc3, Apc5, Apc*6 and *Cul3* etc) were repressed. This suggested that some related protein degradation were down-regulated in *A. nidulans* with ABA induction. The ubiquitin-proteasome pathway may have important role during the ABA signaling transduction^3,4^.

### DEGs involved in nucleotide metabolism and TCA cycle

Nucleotides (purines and pyrimidines) are the building blocks of nucleic acids (DNA and RNA)^35^. They carry chemical energy (ATP and GTP), participate in cell signaling (cGMP and cAMP), and act as enzyme cofactors (CoA, FAD and NADP)^36^. There were 81 out of 219 gene homologs in purine metabolism changed by ABA induction (Table S2). Only 18 gene homologs for enzymes (nucleoside-diphosphate kinase, ribonucleotide reductase, and urease etc) were down-regulated; whereas 63 gene homologs were up-regulated, including enzymes for ribose 5-phosphate (ribose-5P) to aminoimidazole ribotide (AIR), adenine/guanine to xanthine, and dGTP-dAMP energy conversions etc. Meanwhile, about 48 out of 160 gene homologs in pyrimidine metabolism were identified in the DEGs (Table S2). Only 11 gene homologs for enzymes (nucleoside-diphosphate kinase and ribonucleotide reductase etc) were down-regulated; whereas 37 gene homologs for enzymes (rpoA, polA, CTP synthase pyrG, dCMP deaminase, purine-nucleoside phosphorylase, dihydroorotate dehydrogenase etc) were up-regulated. Therefore, nucleotide metabolism pathways in *A. nidulans* were mostly up-regulated by ABA induction.

TCA cycle (citrate cycle) plays a central role in the catabolism of carbohydrates and fatty acids, supplying energy and precursor metabolites^37^. Acetyl-CoA delivers acetyl group to the TCA cycle, linking primary and secondary metabolic pathways^38^. There were 37 out of 120 gene homologs in the TCA cycle changed, all up-regulated by the ABA induction (Table S2). The up-regulated gene homologs are coding for the catalytic enzymes, including phosphoenolpyruvate carboxykinase (*pckA*), pyruvate dehydrogenase (*aceE*), dihydrolipoamide dehydrogenase (*DLD*), malate dehydrogenase (*MDH1*), citrate synthase (*CS*), ATP citrate lyase (*ACLY*), aconitate hydratase (*ACO*), fumarate hydratase (*fumA*), succinate dehydrogenase (*SDHA*), succinyl-CoA synthetase (*LSC1*, *sucD*), dihydrolipoamide succinyltransferase (*DLST*), 2-oxoglutarate dehydrogenase (*OGDH*), and isocitrate dehydrogenase (*IDH3*) etc. The up-regulated TCA cycle will provide energy and precursors for cell growth and secondary metabolism.

### ABA regulates fungal cell cycle and biosynthesis of secondary metabolites

Cell cycle progression goes in sequence through gap (G1) phase, DNA replication (S phase), G2, and mitosis (M phase), which are regulated by various kinases and related protein complex^39^. About 70 out of 268 gene homologs in cell cycle progression were changed by ABA induction (Table S3). Only 13 DEGs were up-regulated, such as homolog genes for condensin complex subunit 3 (*Ycg1*) and regulatory factor X (*Rfx1*) etc. Whereas 57 DEGs were down-regulated, including homolog genes for cell division control protein 7 (*Cdc7*), F-box and leucine-rich repeat protein (*Grr1*), F-box and WD-40 domain protein (*Met30*), mitosis inhibitor protein kinase (*Swe1*), cell cycle arrest protein (*Bub3*), structural maintenance of chromosome 1 (*Smc1*), serine/threonine-protein phosphatase 2A regulatory subunit A (*PP2A*), cell cycle checkpoint protein (*Rad17*), and serine/threonine-protein kinase (*Chk2*) etc. Since the fungal mycelia were harvested during the decline phase, ABA might contribute to the slow down of cell cycle progression.

Genome mining of secondary metabolites and biosynthetic gene clusters in *A. nidulans* FGSC A4 has revealed at least 83 compounds and 56 gene clusters^40^. We have isolated and identified 34 natural products, including 13 new compounds, from *A. nidulans* MA-143^17,18^. Comparative transcriptome analysis indicated that several biosynthetic pathways for secondary metabolites were significantly changed by ABA induction, including biosynthesis of anthraquinones, terpenoids, and steroids etc (Fig. S8). Sterigmatocystin is one of the intermediates of aflatoxins, which are highly toxic mycotoxins^41^. About 8 out of 22 genes in the sterigmatocystin gene cluster were down-regulated by ABA induction (Table S2), including homolog genes for norsolorinic acid ketoreductase (*aflD*), versiconal hemiacetal acetate esterase (*ESTA*), and demethylsterigmatocystin 6-O-methyltransferase (*dmtA*) etc. This suggests that the sterigmatocystin production might be reduced by ABA induction, while some intermediate products might be increased.

Fungal terpenoids were synthesized by the MVA pathway for the building blocks: isopentenyl diphosphate (IPP). There were 11 out of 55 genes in terpenoid backbone biosynthesis up-regulated by ABA induction (Fig. S8), including homolog genes for hydroxymethylglutaryl-CoA reductase (*HMGCR*), phosphomevalonate kinase (*mvaK2*), farnesyl diphosphate synthase (*FDPS*), geranylgeranyl diphosphate synthase (*GGPS1*), and hexaprenyl-diphosphate synthase (*hexPS*) etc. These may suggest enhanced production of geranyl diphosphate (GPP), farsenyl diphosphate (FPP), geranylgeranyl diphosphate (GGPP), and hexaprenyl diphosphate (HPP), which are the precursors for biosynthesis of ubiquinones and other terpenoid-quinones.

Steroids are lipid components of cell membranes and also function as signaling molecules^42^. There were 36 out of 107 genes in steroid biosynthesis changed by ABA induction (Table S2). Only 9 gene homologs were down-regulated, including genes for sterol-4α-carboxylate 3-dehydrogenase (*ERG26*), 3-keto steroid reductase (*ERG27*), and cholestenol Δ-isomerase (*EBP*) etc; whereas 27 gene homologs were up-regulated, such as genes for farnesyl-diphosphate farnesyltransferase (*FDFT1*), squalene monooxygenase (*ERG1*), Δ14-sterol reductase (*ERG24*), Δ7-sterol 5-desaturase (*ERG3*), sterol 24-C-methyltransferase (*ERG6*), C-8 sterol isomerase (*ERG2*), sterol 22-desaturase (*ERG5*), and sterol O-acyltransferase (*SOAT*) etc. These suggest that the ergosterol (provitamin D2) production may be up-regulated. These metabolites might contribute to the biosynthesis of phytosterols.

Based on the KEGG enrichment of DEGs by ABA induction, schematic representation of the main metabolic pathways differentially regulated is shown in Fig. S8. Some other metabolic pathways, such as metabolism of nitrogen, amino acid, lipid and sugar, were also partially affected by ABA induction. Metabolic profiles analysis indicated that production of secondary metabolites was changed by ABA induction as detected by HPLC (Fig. 2). Our lab has constructed a library of fungal natural products, and we have isolated and identified 34 different natural products from *A. nidulans* MA-143^17,18^. After compared with the compound library by UV spectra and retention time, it indicated that productions of anthraquinones were changed with ABA induction. Production of sterigmatocystin, secosterigmatocystin, and averufin were slightly reduced; while production of vericolorin B, paeciloquinone E, norsolorinic acid, and a new compound were slightly increased. Bioactivity tests indicated that these natural products have varying insect-resistance and anti-pathogen activities^43–45^. Interestingly, the norsolorinic acid, which may occur as phytoalexin, was also isolated from some plant^46^. The physiological function of these natural products during plant-microbe interaction still needs further study. It’s speculated that ABA may induce endophytic fungi to produce specific metabolites, which might be beneficial to the host plant for environmental adaption.

## Discussion

Microbial biosyntheses of plant hormones have been reported in diverse species^47^. For example, the most widely distributed auxin IAA was also produced by plant-associated bacteria and fungi^9,48^. The biosynthesis and regulation of IAA in pathogenic and symbiotic bacteria, such as *Agrobacterium, Pseudomonas, Rhizobium, Bradyrhizobium*, *Bacillus, Azotobacter*, *Azospirillum*, and *Sulfitobacter*, has been well studied^49^. Although the biosynthetic pathways evolved independently in microbes and plants, IAA may play vital role during the plant-microbe signaling^5^. Microbial production of IAA is involved in physiological processes from plant pathogenesis to growth promotion^1,9^. In *Saccharomyces cerevisiae*, IAA (50 μM) induced invasive fungal growth, adhesion and filamentation by a specific transcription factor Yap1^8^. Whereas, higher concentration of IAA (200 μM) inhibited the growth of many plant-associated bacteria (such as *Agrobacterium*) and inactivated the virulence gene expression^50^. In marine ecosystems, IAA synthesized by *Sulfitobacter* promoted cell division of diatom. IAA acts as signaling molecule in the diatom-bacteria interactions, which needs exchange of nutrients, like organosulfur, ammonia, and tryptophan^1^. This hormone signaling mode might be prevalent during plant-microbe interactions.

Some phytohormones were originally isolated from fungi, and finally proved to be plant hormone^2,51^. For instance, GAs were firstly isolated from a rice pathogen *Gibberella fujikuroi*, latter found to stimulate rice root growth, and finally identified as natural regulators of plant growth and development^51^. The GA biosynthetic pathways are different in fungi, bacteria and plants, which may have evolved independently^51,52^. SLs were first found to induce hyphal branching of arbuscular mycorrhizal fungus *Gigaspora margarita*, and latter proved to be a new plant hormone inhibiting shoot branching^6,53^. ABA was also synthesized by some plant pathogenic fungi, including *Botrytis cinerea*, *Cercospora rosicola*, *Fusarium oxysporum*, and *Rhizoctonia solani* etc^10^. *B. cinerea* is used for commercial production of ABA, which is synthesized by the MVA pathway via FPP intermediates; whereas plants utilize the MEP pathway and carotenoids as intermediates^12,13^. Besides the central ABA biosynthetic pathway, genes encoding polysaccharide hydrolases, sugar transporters, and precursor acetyl-CoA also contribute to the ABA biosynthesis in fungi^13^.

**Figure 5:**
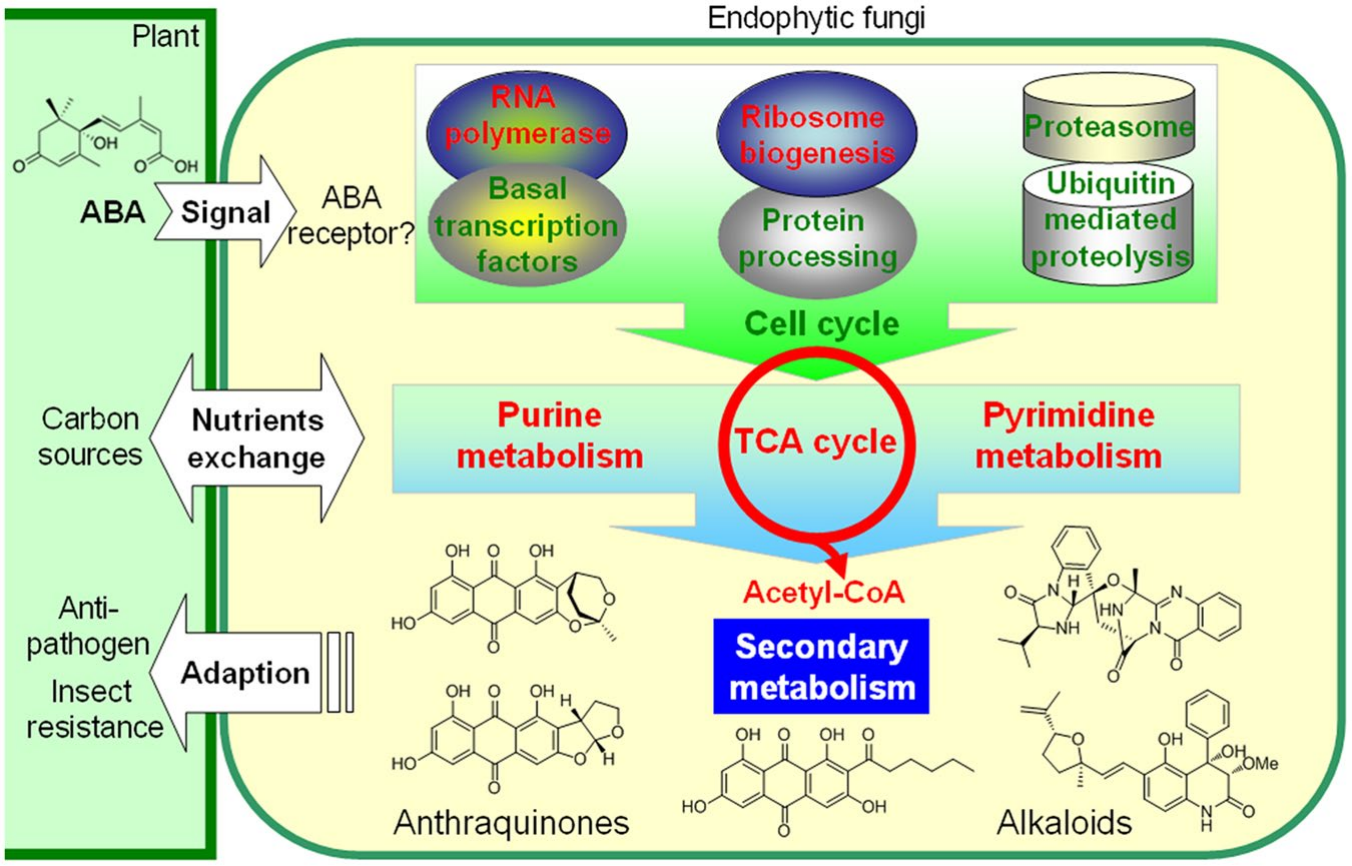
Proposed cross-talk between plant and endophytic fungus *A. nidulans* via ABA. The plant ABA might be sensed by endophytic fungus, which may change the gene transcription, protein biosynthesis and degradation. Then, primary metabolism may changed, which could exchange nutrients with host cell. Fungal secondary metabolites may also be changed for their environmental adaption.

In summary of these transcriptome and metabolite analyses, we proposed an interaction model between plant and endophytic fungi mediated by ABA (Fig. 5). The plant hormone ABA might be sensed by some receptor in endophytic fungi. The central cellular machines for gene transcription, protein synthesis and degradation were changed. Then it may affect the nucleotide metabolism, which might exchange nutrients and energy with plant cell. The TCA cycle was also up-regulated by ABA induction. By the main precursor acetyl-CoA, the biosynthesis of secondary metabolites was also altered. Production of mycotoxins, which have insect-resistance and anti-pathogen activity, might contribute to adaption of host plant cell against environmental stresses. These interactions are mediated by hormone signaling, nutrients exchange, and secondary metabolites. The ranges of responses to ABA in *Aspergillus* are quite interesting, and especially the effects on transcription, translation, glycolysis, and cell proliferation might indicate an effect on an mTOR signaling pathway^54,55^. Recently, it’s reported that the plant hormone cytokinin was specifically sensed by a bacterial receptor histidine kinase, which promoted plant adaptation to oxidative stress through phosphorelay and second messenger (c-di-GMP)^56^. The molecular mechanism involving ABA receptor and the signal transduction pathway during host-microbe interactions still needs further investigations.

It’s reported that higher level of IAA only inhibits the growth of plant-associated bacteria, but not these non-plant-associated bacteria from other ecological niches^50^. The cross-talk between plant and associated bacteria might be developed during long term of co-evolution^1^. It would be interesting to compare their genetic and transcriptomic differences, and reveal the underlying molecular hormone signaling mechanism. The plant hormone ABA affects not only fungal growth and development, but also primary and secondary metabolisms of endophytic fungus *A. nidulans*. ABA not just acts as hormone in plants, but also as signaling molecule in fungi^15^. It’s demonstrated that ABA may promote fungal growth and have negative effects on plant pathogen resistance^57,58^. Moreover, ABA may also regulate the immune modulation and glucose homeostasis in mammals, which might have some medical applications^59,60^. The phytohormone ABA may act as plant pathogen effector and mammal immune regulator, which are mediated by microbes^15^. As an ancient molecule, ABA might function as interspecies communication signal in the natural ecosystems^10^.

## Methods

### Fungal strain and culture conditions

The endophytic fungus *Aspergillus nidulans* strain MA-143 (GenBank accession no. JQ839285) was originally isolated from the fresh leaves of mangrove plant^18,61^. The fungal strain was grown on potato dextrose agar (PDA) plates at 28 °C for 5 ~ 10d for conidial suspensions. For macro-morphological observations, the isolates were inoculated on agar plates and incubated at 28 °C in the dark for 5~10d. For micro-morphological observations, fungal materials were examined using light microscopy (ZEISS Imager A2). Conidial suspensions (10^6^ spores·mL^−1^) were inoculated into yeast extract sucrose (YES) medium (yeast extract 2%, sucrose 15%, MgSO_4_·7H_2_O 0.05%, and sea water 50%), incubated at 28 °C 200 rpm on an orbital shaker for 1d as seed culture^62^. The seed culture (1%) was inoculated into 500 mL flask containing 150 mL ICI medium (glucose 2%, NaNO_3_ 0.1%, MgSO_4_·7H_2_O 0.1%, KH_2_PO_4_ 0.1%, ZnSO_4_ 0.01%, and sea water 50%) for fermentation at 28 °C at 200 rpm, and harvested at different time (2, 4, 6, 8, and 10d)^63^. The ABA induction concentrations were as follows: 10~100 nM and 10~100 μM. ABA was dissolved in ethanol as stock solution and added by 0.1%. All experiments were in triplicates with the mean values ± standard deviations (SD) shown.

### Sample collection and library preparation

Samples from three flasks were mixed and vacuum-filtered. The culture filtrates were extracted by ethyl acetate (1:1) and analyzed by HPLC (Dionex) with C18 column (4.6 × 250 mm, 5 μm) and UV detector (200~600 nm). The metabolites extraction and HPLC analysis were following previous procedures^64^. Isolation and identification of natural products from *A. nidulans* were following protocols as described previously^17,18,61^. The mycelium pellets were dried by filter paper, weighed, and frozen in liquid nitrogen for total RNA extraction with Fungal RNA Miniprep Kit (OMEGA). RNA concentration and integrity were measured using Qubit 2.0 Flurometer (Life Technologies) and Bioanalyzer 2100 system (Agilent Technologies). Transcriptome sequencing libraries were prepared using NEBNext Ultra RNA Library Prep Kit (NEB, USA) for Illumina following manufacturer’s protocols^13^.

### Transcriptome sequencing and RNA-seq data analysis

The transcriptome library preparations were sequenced using the Illumina Hiseq PE150 platform (Illumina, USA) by the Novogene (Beijing, China). RNA-seq data were deposited in the NCBI Sequence Read Archive (SRA) database under the BioProject PRJNA445751. For quality control, clean data were obtained from raw reads (150 bp paired-end) with Q20, Q30 and GC content calculated. Transcriptome reconstruction from RNA-seq without a genome was performed with the Trinity^19^. Unigenes were defined as longer transcripts from sequence clustering of contigs, which were combined certain long reads without N^65^. For quantification of gene expression level, FPKM (Fragments Per Kilobase of transcript sequence per Millions base pairs) values were calculated by the Cufflinks (2.1.1)^20^. Differential expression gene analyses of two conditions were performed using the DESeq R package (1.18.0)^66^. P-values were adjusted by the Benjamini & Hochberg’s method. DEGs were selected with an adjusted P-value < 0.05. Functional classification of DEGs was performed by GO enrichment analysis using the GOseq R package^67^ with corrected P-value < 0.05. Enrichment of DEGs in the KEGG metabolic pathways was analyzed by the KOBAS software^68^. Transcriptional changes in abundance (P-value < 0.05) were used to identify the significant DEGs in *A. nidulans* with ABA induction compared to controls.

## Electronic supplementary material

Supplementary Information
