# Supplementary material for: The plant hormone abscisic acid regulates the growth and metabolism of endophytic fungus Aspergillus nidulans
Source: Sci Rep. 2018 Apr 25;8:6504. doi: 10.1038/s41598-018-24770-9 (PMC5916901; doi:10.1038/s41598-018-24770-9)
Supplement: Supplementary file 1 — Supplementary Information [file 41598_2018_24770_MOESM1_ESM.pdf]

The plant hormone abscisic acid regulates the growth and metabolism of endophytic fungus *Aspergillus nidulans*

Gangming Xu[1,†,*], Suiqun Yang[1,2,†], Linghong Meng[1] & Bin-Gui Wang[1,*]

[1]Key Laboratory of Experimental Marine Biology, Institute of Oceanology, Chinese Academy of Sciences; Laboratory of Marine Biology and Biotechnology, Qingdao National Laboratory for Marine Science and Technology; Nanhai Road 7, Qingdao 266071, People's Republic of China.

[2]University of Chinese Academy of Sciences, 19A Yuquan Road, Beijing 100049, P. R. China.

[*]Correspondence should be addressed to B.-G.W. (wangbg@ms.qdio.ac.cn) and G.M.X. (aericxu@gmail.com).

[†]These authors contributed equally to this work.

Supplementary Information

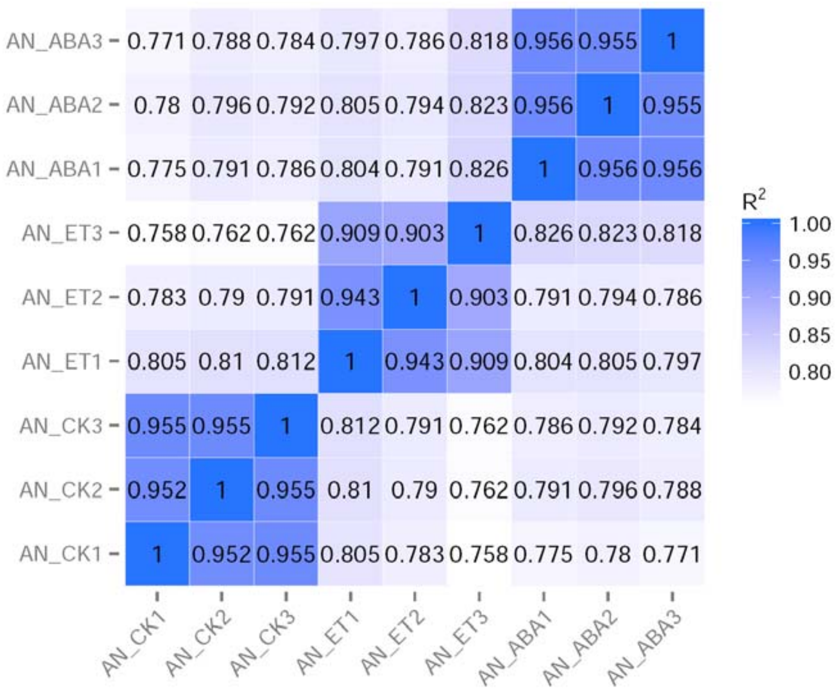

Figure S1. Pearson correlations between samples.

A. nidulans (AN_CK), *A. nidulans* with 0.1% ethanol (AN_ET), and *A. nidulans* with 100 nM ABA (AN_ABA).

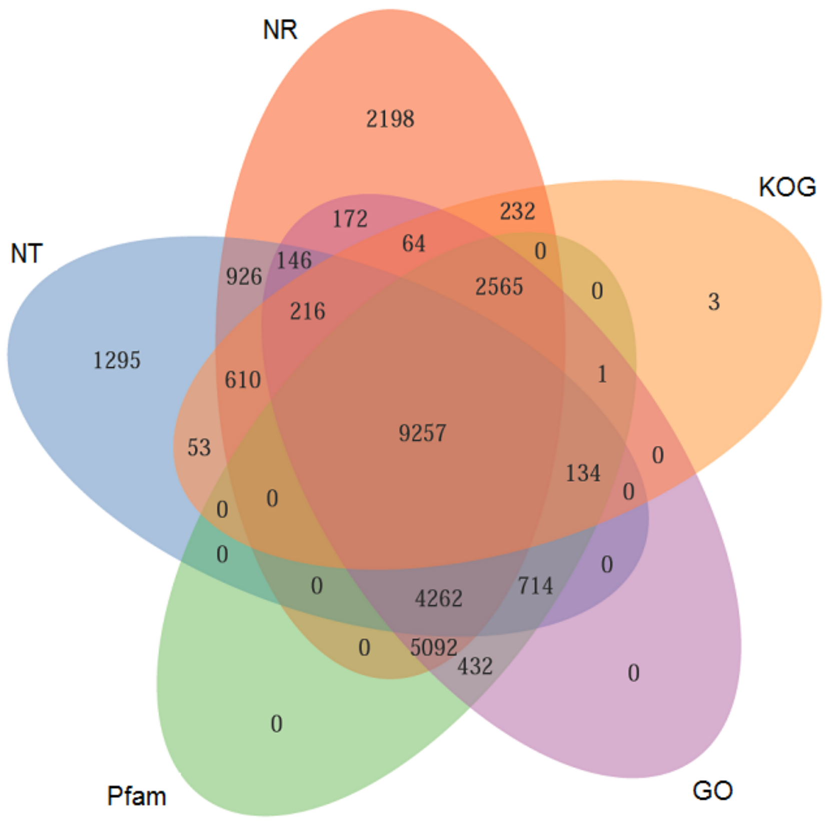

Figure S2. Gene functional annotation in 5 databases.

NR (NCBI non-redundant protein sequences), NT (NCBI nucleotide sequences), Pfam (Protein family), GO (Gene Ontology), KOG (euKaryotic Ortholog Groups).

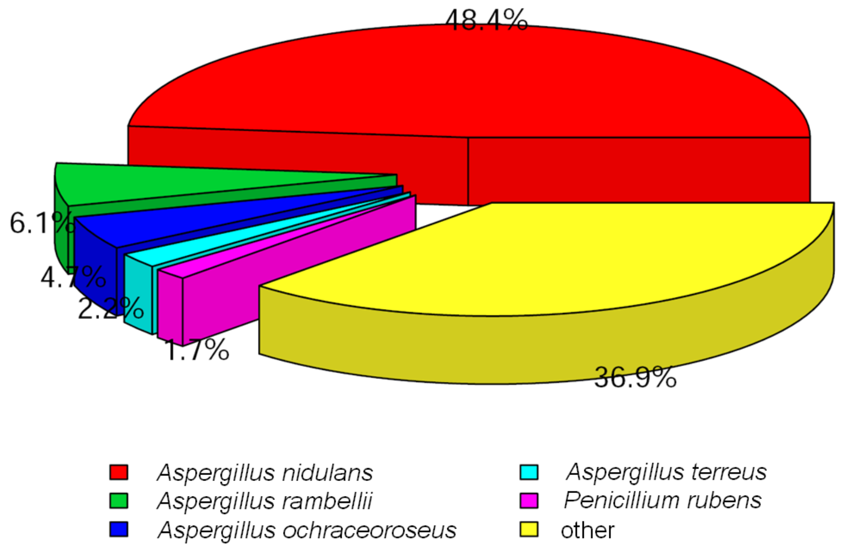

Figure S3. Species classification after gene functional annotation.

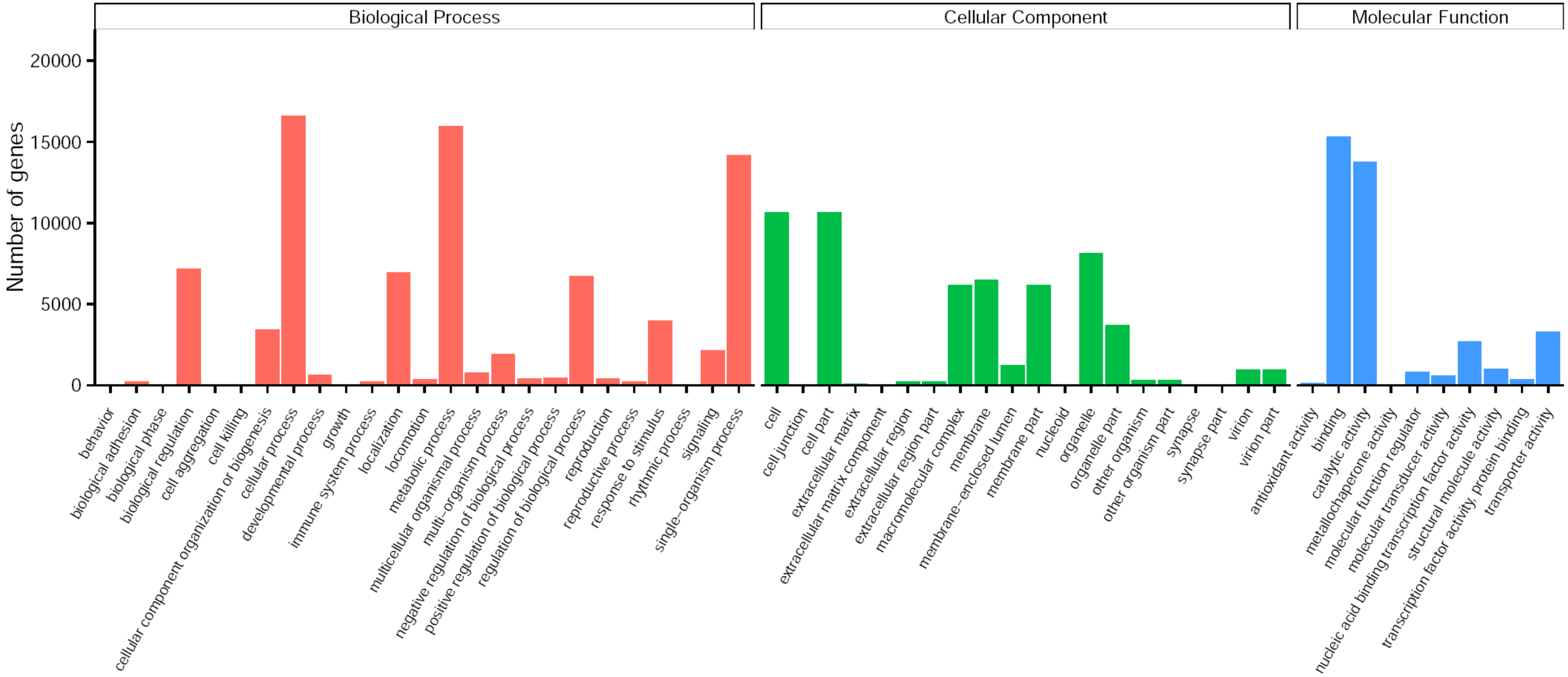

Figure S4. Total annotated gene classification in the GO database.

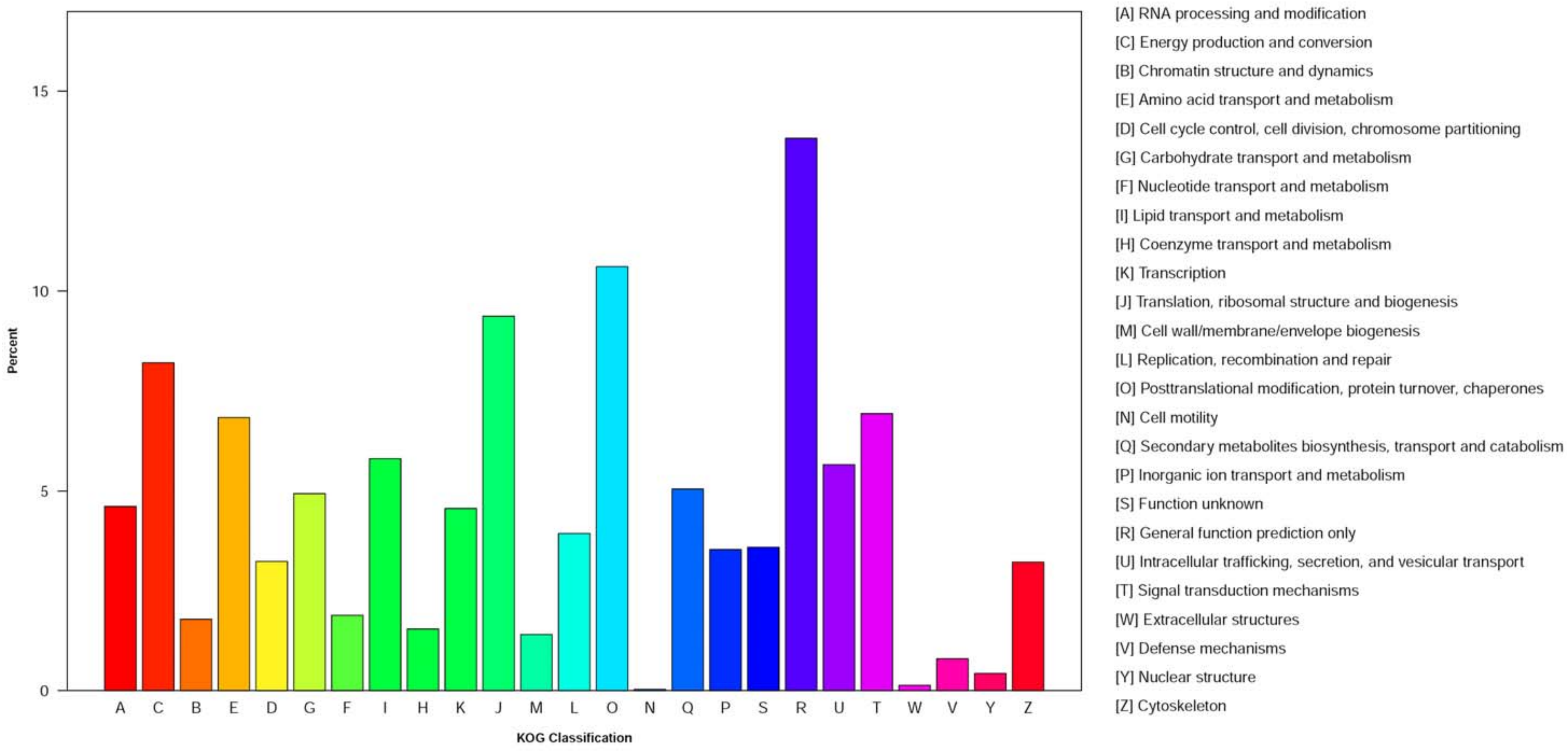

Figure S5. Total annotated gene classification in the KOG database.

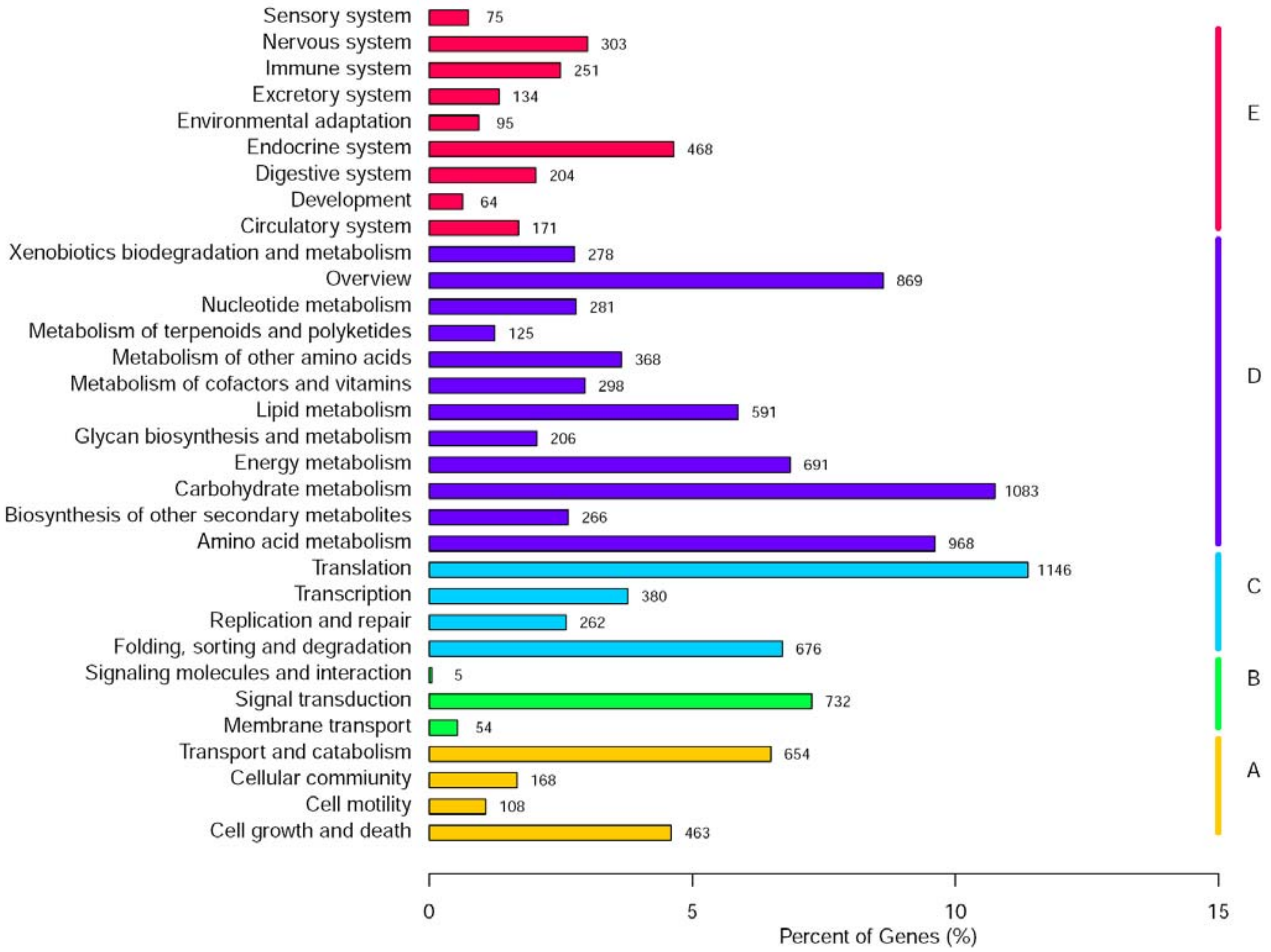

Figure S6. Total annotated gene classification in the KEGG database.

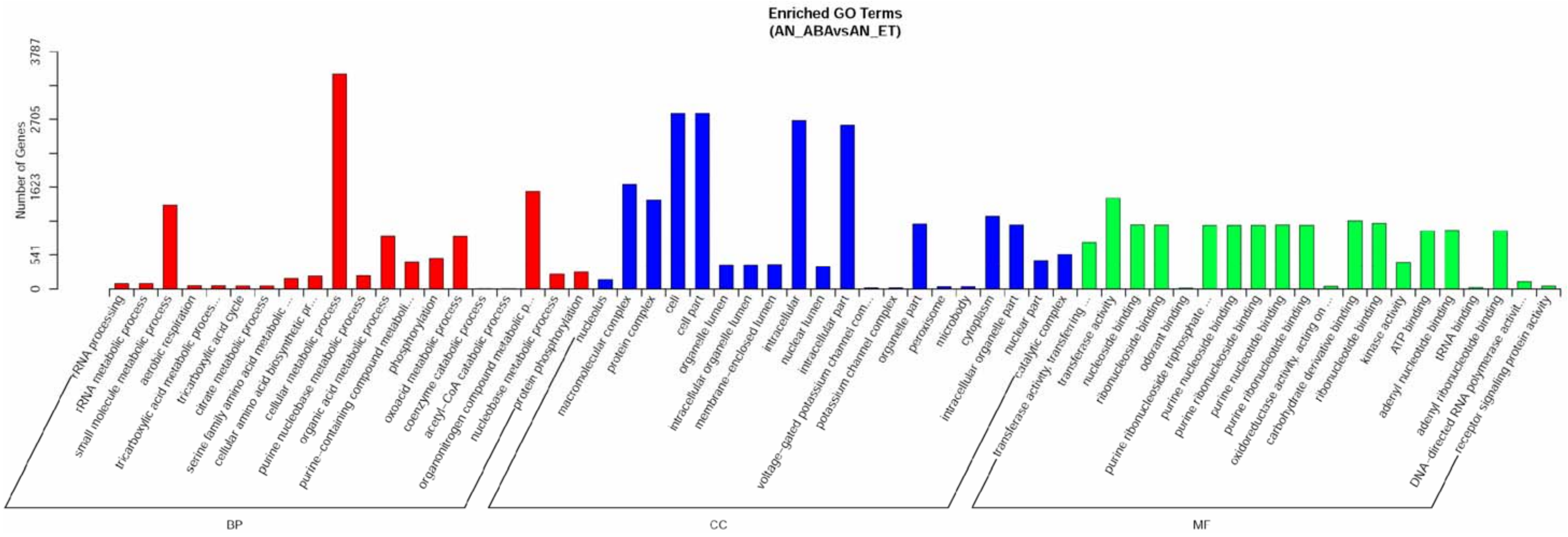

Figure S7. GO enrichment of differentially expressed genes (DEGs).

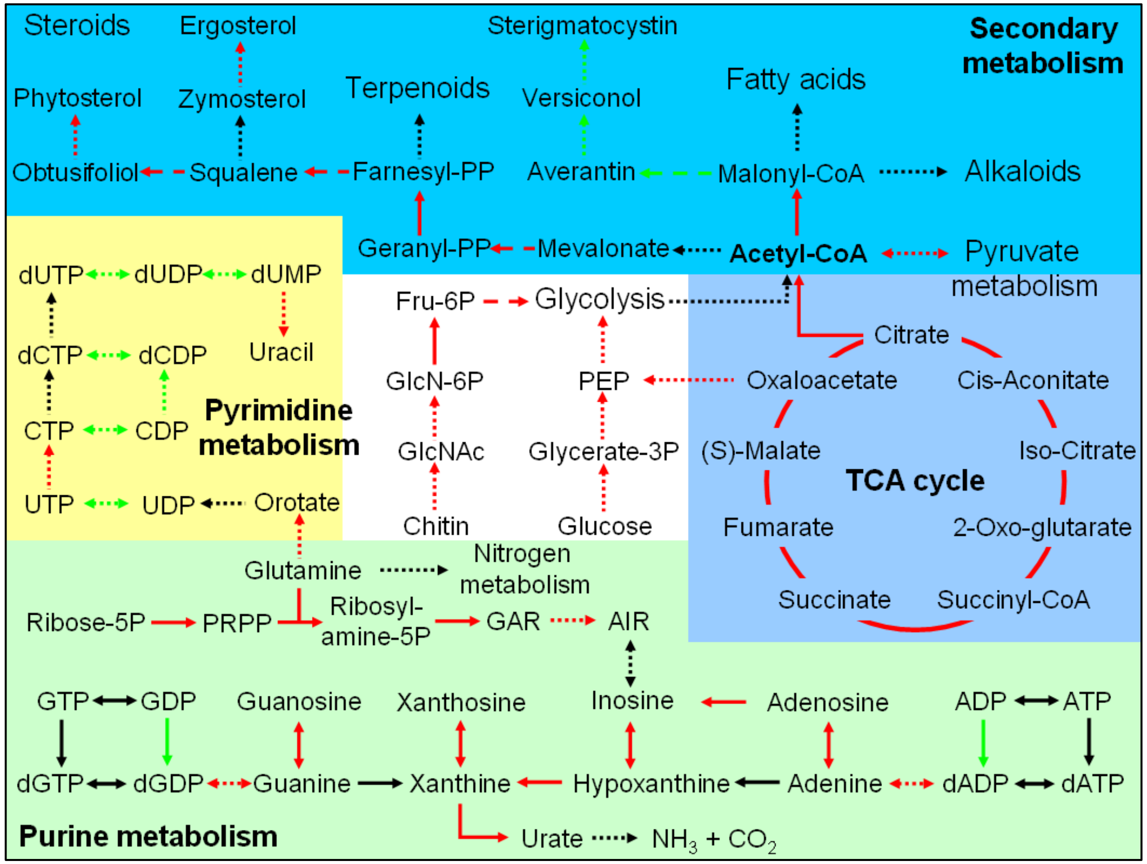

Figure S8. Main metabolic pathways of *A. nidulans* in response to ABA.

Some of the main metabolic pathways are shown based on the KEGG enrichment of DEGs by ABA induction. Red: up-regulated, green: down-regulated, black: not significant, dash line: multiple steps.

Table S1. Quality control summary of RNA-seq clean data

Sample	Raw reads	Clean reads	Clean bases	Error (%)	Q20 (%)	Q30 (%)	GC content (%)
AN_CK1	61534468	59293988	8.89G	0.03	94.88	88.11	52.15
AN_CK2	51409162	49573308	7.44G	0.03	94.87	88.09	52.13
AN_CK3	57485434	55408742	8.31G	0.03	94.85	88.06	52.26
AN_ET1	53049054	51064850	7.66G	0.03	94.7	87.78	52.27
AN_ET2	52382910	50424830	7.56G	0.03	94.7	87.8	52.35
AN_ET3	46400902	44289682	6.64G	0.02	95.9	89.81	52.17
AN_ABA1	46132620	45151138	6.77G	0.02	95.86	89.62	52.28
AN_ABA2	46791992	45784908	6.87G	0.02	95.83	89.59	52.3
AN_ABA3	48200838	47228770	7.08G	0.02	95.88	89.66	52.29

Note: *A. nidulans* (AN_CK), *A. nidulans* with 0.1% ethanol (AN_ET), and *A. nidulans* with 100 nM ABA (AN_ABA).

Table S2. The most enriched KEGG pathway terms up-regulated by ABA

Term	Sample number	Background number	P-value	Corrected P-value	UniGenes	KO	Gene name
Ribosome biogenesis in eukaryotes	62	150	5.42336579525e-13	5.42336579525e-11	Cluster-4423.17831<br>Cluster-4423.17830<br>Cluster-4423.16922<br>Cluster-4423.12760<br>Cluster-4423.14733<br>Cluster-4423.13834<br>Cluster-4423.9982<br>Cluster-4423.9707<br>Cluster-4423.16761<br>Cluster-4423.16324<br>Cluster-4423.14066<br>Cluster-4423.14826<br>Cluster-4423.15808<br>Cluster-4423.13584<br>Cluster-4423.19143<br>Cluster-4423.15369<br>Cluster-4423.16691<br>Cluster-4423.15742<br>Cluster-4423.17066<br>Cluster-4423.15119<br>Cluster-4423.16407	K14538<br>K14538<br>K14559<br>K14556<br>K14545<br>K14573<br>K14545<br>K03685<br>K11128<br>K14548<br>K14568<br>K14554<br>K14566<br>K14558<br>K14557<br>K11128<br>K11131<br>K14563<br>K14758<br>K11129<br>K14758	*NUG1, GNL3 NUG1,*<br>*GNL3 MPP10 DIP2,*<br>*UTP12, WDR3 RRP7*<br>*NOP4, RBM28 RRP7*<br>*rnc, DROSHA, RNT1*<br>*GAR1, NOLA1 UTP4,*<br>*CIRH1A EMG1, NEP1*<br>*UTP21, WDR36*<br>*UTP24, FCF1 PWP2,*<br>*UTP1 UTP6 GAR1,*<br>*NOLA1 DKC1, NOLA4,*<br>*CBF5 NOP1, FBL*<br>*HRR25 NHP2, NOLA2*<br>*HRR25 NUG2, GNL2*<br>*NOB1 NUG2, GNL2*<br>*RRP7 NOP10, NOLA3*<br>*NUG1, GNL3 NAN1,*<br>*UTP17, WDR75 NMD3*<br>*UTP24, FCF1 RCL1*<br>*UTP15 UTP21, WDR36*<br>*NOP58 PWP2, UTP1*

					Cluster-4423.16810<br>Cluster-4423.15245<br>Cluster-4423.15489<br>Cluster-4423.14368<br>Cluster-4423.16738<br>Cluster-4423.18246<br>Cluster-4423.16753<br>Cluster-4423.12543<br>Cluster-4423.12553<br>Cluster-4423.14288<br>Cluster-4423.12077<br>Cluster-4423.13354<br>Cluster-4423.17331<br>Cluster-4423.11808<br>Cluster-4423.17263<br>Cluster-4423.18160<br>Cluster-4423.16714<br>Cluster-4423.15729<br>Cluster-4423.16318<br>Cluster-4423.16330<br>Cluster-4423.14950<br>Cluster-4423.18517<br>Cluster-4423.13483<br>Cluster-4423.16924<br>Cluster-4423.15393<br>Cluster-4423.16769	K14537<br>K11883<br>K14537<br>K14545<br>K11130<br>K14538<br>K14552<br>K07562<br>K14566<br>K11108<br>K14549<br>K14554<br>K14565<br>K14558<br>K14546<br>K14554<br>K14538<br>K11128<br>K14550<br>K14569<br>K14575<br>K14538<br>K14567<br>K14559<br>K14537<br>K14563	*UTP5, WDR43 UTP21, WDR36 NUG1, GNL3 GAR1, NOLA1 UTP10, HEATR1 BMS1 AFG2, DRG1, SPATA5 NUG1, GNL3 UTP14 MPP10 NUG2, GNL2 NOP1, FBL HRR25 NXF, TAP, MEX67 RAN UTP14 XRN2, RAT1 NAT10, KRE33 RIOK2 AFG2, DRG1, SPATA5 AFG2, DRG1, SPATA5 NHP2, NOLA2 LSG1 HRR25 NOG1 NAT10, KRE33 UTP21, WDR36*

					Cluster-4423.16241<br>Cluster-4423.14199<br>Cluster-4423.17981<br>Cluster-4423.16412<br>Cluster-4423.15694<br>Cluster-4423.17096<br>Cluster-4423.8558<br>Cluster-4423.8562<br>Cluster-4423.16991<br>Cluster-4423.18231<br>Cluster-4423.12748<br>Cluster-4423.15234<br>Cluster-4423.15493<br>Cluster-4423.16577<br>Cluster-4423.14117	K14758<br>K14284<br>K07936<br>K14567<br>K12619<br>K14521<br>K07179<br>K14575<br>K14575<br>K11129<br>K14539<br>K14758<br>K06943<br>K14521<br>K14554	
Purine metabolism	63	219	5.87950606023e-08	2.93975303011e-06	Cluster-4423.18833<br>Cluster-4423.18481<br>Cluster-4423.18589<br>Cluster-4423.14304<br>Cluster-4423.14539<br>Cluster-4423.8068<br>Cluster-4423.15794<br>Cluster-4423.7722<br>Cluster-4423.9494<br>Cluster-4423.12170<br>Cluster-4423.16349	K03783<br>K00873<br>K03011<br>K00764<br>K01487<br>K01466<br>K01952<br>K03023<br>K00948<br>K03014<br>K00365	*punA PK, pyk RPB3,*<br>*POLR2C purF, PPAT*<br>*E3.5.4.3, guaD allB*<br>*purL, PFAS RPC3,*<br>*POLR3C PRPS, prsA*<br>*RPB6, POLR2F uaZ*<br>*XDH APA1_2 RPC40,*<br>*POLR1C RPA1,*<br>*POLR1A RPC1,*<br>*POLR3A APRT, apt*<br>*punA RPB6, POLR2F*

					Cluster-4423.17306<br>Cluster-4423.7603<br>Cluster-4423.10692<br>Cluster-4423.16116<br>Cluster-4423.17584<br>Cluster-4423.17142<br>Cluster-4423.15116<br>Cluster-4423.14449<br>Cluster-4423.15073<br>Cluster-4423.16464<br>Cluster-4423.12171<br>Cluster-4423.11664<br>Cluster-4423.11515<br>Cluster-4423.13147<br>Cluster-4423.16851<br>Cluster-4423.15660<br>Cluster-4423.14405<br>Cluster-4423.15180<br>Cluster-4423.10100<br>Cluster-4423.15974<br>Cluster-4423.20544<br>Cluster-4423.6904<br>Cluster-4423.6905<br>Cluster-4423.12764<br>Cluster-4423.17811<br>Cluster-4423.15460	K00106<br>K00988<br>K03027<br>K02999<br>K03018<br>K00759<br>K03783<br>K03014<br>K00948<br>K01466<br>K03014<br>K03783<br>K03014<br>K02684<br>K00948<br>K00873<br>K01490<br>K03023<br>K00958<br>K00106<br>K03014<br>K03022<br>K03022<br>K03002<br>K03783<br>K03019	*PRPS, prsA allB RPB6, POLR2F punA RPB6, POLR2F PRI1 PRPS, prsA PK, pyk AMPD RPC3, POLR3C sat, met3 XDH RPB6, POLR2F RPC8, POLR3H RPC8, POLR3H RPA2, POLR1B punA RPC11, POLR3K RPA2, POLR1B PRPS, prsA sat, met3 RPB1, POLR2A XDH ADE5 ylbA, UGHY, RPC19, POLR1D CECR1, ADA2 allB RPB12, POLR2K ADE2 RPC2, POLR3B RPA1, POLR1A AMPD add, ADA XDH RPA43 allB RPA2, POLR1B PRPS, prsA PRPS, prsA PRPS, prsA PK, pyk purF, PPAT RPA49, POLR1E*

					Cluster-4423.11999<br>Cluster-4423.17020<br>Cluster-4423.9201<br>Cluster-4423.14629<br>Cluster-4423.16721<br>Cluster-4423.13256<br>Cluster-4423.16314<br>Cluster-4423.18829<br>Cluster-4423.19975<br>Cluster-4423.17339<br>Cluster-4423.15416<br>Cluster-4423.14192<br>Cluster-4423.7538<br>Cluster-4423.16534<br>Cluster-4423.17763<br>Cluster-4423.13957<br>Cluster-4423.20053<br>Cluster-4423.14607<br>Cluster-4423.17192<br>Cluster-4423.8589<br>Cluster-4423.14229<br>Cluster-4423.18310<br>Cluster-4423.13687<br>Cluster-4423.16096<br>Cluster-4423.16841<br>Cluster-4423.16998	K03002<br>K00948<br>K00958<br>K03006<br>K00106<br>K11788<br>K14977<br>K03020<br>K19572<br>K01466<br>K03009<br>K11808<br>K03021<br>K02999<br>K01490<br>K01488<br>K00106<br>K03004<br>K01466<br>K03002<br>K00948<br>K00948<br>K00948<br>K00873<br>K00764<br>K03005	

Citrate cycle (TCA cycle)	37	120	8.81541568422e-06	0.000293847189474	Cluster-4423.12885<br>Cluster-4423.18583<br>Cluster-4423.15591<br>Cluster-4423.17113<br>Cluster-4423.18115<br>Cluster-4423.14978<br>Cluster-4423.14618<br>Cluster-4423.12109<br>Cluster-4423.16038<br>Cluster-4423.12863<br>Cluster-4423.15409<br>Cluster-4423.15263<br>Cluster-4423.17406<br>Cluster-4423.16981<br>Cluster-4423.11475<br>Cluster-4423.16947<br>Cluster-4423.12541<br>Cluster-4423.17408<br>Cluster-4423.17287<br>Cluster-4423.12893<br>Cluster-4423.13547<br>Cluster-4423.14312<br>Cluster-4423.17001<br>Cluster-4423.22170<br>Cluster-4423.22232<br>Cluster-4423.15285	K00026<br>K01679<br>K01679<br>K01647<br>K00162<br>K00030<br>K01648<br>K01647<br>K01648<br>K00234<br>K01681<br>K00161<br>K01647<br>K01610<br>K01610<br>K01647<br>K00234<br>K01647<br>K01610<br>K01900<br>K00234<br>K00164<br>K01648<br>K00161<br>K00161<br>K00382	*MDH2 E4.2.1.2B, fumC E4.2.1.2B, fumC CS, gltA PDHB, pdhB IDH3 ACLY CS, gltA ACLY SDHA, SDH1 ACO, acnA PDHA, pdhA CS, gltA E4.1.1.49, pckA E4.1.1.49, pckA CS, gltA SDHA, SDH1 CS, gltA E4.1.1.49, pckA LSC2 SDHA, SDH1 OGDH, sucA ACLY PDHA, pdhA PDHA, pdhA DLD, lpd, pdhD DLST, sucB DLD, lpd, pdhD E4.1.1.49, pckA SDHB, SDH2 ACLY CS, gltA ACLY DLST, sucB OGDH, sucA CS, gltA DLST, sucB*

					Cluster-4423.19255<br>Cluster-4423.15143<br>Cluster-4423.17157<br>Cluster-4423.10545<br>Cluster-4423.17012<br>Cluster-4423.15059<br>Cluster-4423.14782<br>Cluster-4423.11549<br>Cluster-4423.19569<br>Cluster-4423.17432<br>Cluster-4423.14399	K00658<br>K00382<br>K01610<br>K00235<br>K01648<br>K01647<br>K01648<br>K00658<br>K00164<br>K01647<br>K00658	
RNA polymerase	24	61	1.41837465175e-05	0.000354593662938	Cluster-4423.18589<br>Cluster-4423.12764<br>Cluster-4423.7722<br>Cluster-4423.12171<br>Cluster-4423.12170<br>Cluster-4423.10692<br>Cluster-4423.16116<br>Cluster-4423.17584<br>Cluster-4423.11515<br>Cluster-4423.15180<br>Cluster-4423.20544<br>Cluster-4423.6904<br>Cluster-4423.6905<br>Cluster-4423.15460<br>Cluster-4423.11999	K03011<br>K03002<br>K03023<br>K03014<br>K03014<br>K03027<br>K02999<br>K03018<br>K03014<br>K03023<br>K03014<br>K03022<br>K03022<br>K03019<br>K03002	*RPB3, POLR2C RPA2, POLR1B RPC3, POLR3C RPB6, POLR2F RPB6, POLR2F RPC40, POLR1C RPA1, POLR1A RPC1, POLR3A RPB6, POLR2F RPC3, POLR3C RPB6, POLR2F RPC8, POLR3H RPC8, POLR3H RPC11, POLR3K RPA2, POLR1B RPB1,*

					Cluster-4423.14629<br>Cluster-4423.18829<br>Cluster-4423.15416<br>Cluster-4423.7538<br>Cluster-4423.16534<br>Cluster-4423.14607<br>Cluster-4423.8589<br>Cluster-4423.14449<br>Cluster-4423.16998	K03006<br>K03020<br>K03009<br>K03021<br>K02999<br>K03004<br>K03002<br>K03014<br>K03005	*POLR2A RPC19, POLR1D RPB12, POLR2K RPC2, POLR3B RPA1, POLR1A RPA43 RPA2, POLR1B RPB6, POLR2F RPA49, POLR1E*
Pyrimidine metabolism	37	160	0.00119940063452	0.0239880126904	Cluster-4423.18833<br>Cluster-4423.18589<br>Cluster-4423.12764<br>Cluster-4423.7722<br>Cluster-4423.12171<br>Cluster-4423.12170<br>Cluster-4423.8781<br>Cluster-4423.15637<br>Cluster-4423.10692<br>Cluster-4423.16116<br>Cluster-4423.17584<br>Cluster-4423.15116<br>Cluster-4423.15323<br>Cluster-4423.17811<br>Cluster-4423.11515<br>Cluster-4423.13147<br>Cluster-4423.10202	K03783<br>K03011<br>K03002<br>K03023<br>K03014<br>K03014<br>K01493<br>K01937<br>K03027<br>K02999<br>K03018<br>K03783<br>K01956<br>K03783<br>K03014<br>K02684<br>K01493	*punA RPB3, POLR2C RPA2, POLR1B RPC3, POLR3C RPB6, POLR2F RPB6, POLR2F comEB pyrG, CTPS RPC40, POLR1C RPA1, POLR1A RPC1, POLR3A punA carA, CPA1 punA RPB6, POLR2F PRI1 comEB pyrG, CTPS RPC3, POLR3C RPB6, POLR2F RPC8, POLR3H RPC8, POLR3H punA RPC11, POLR3K RPA2, POLR1B RPB1,*

					Cluster-4423.14289<br>Cluster-4423.15180<br>Cluster-4423.20544<br>Cluster-4423.6904<br>Cluster-4423.6905<br>Cluster-4423.11664<br>Cluster-4423.15460<br>Cluster-4423.11999<br>Cluster-4423.14629<br>Cluster-4423.18829<br>Cluster-4423.15416<br>Cluster-4423.13169<br>Cluster-4423.7538<br>Cluster-4423.16534<br>Cluster-4423.19081<br>Cluster-4423.14607<br>Cluster-4423.13051<br>Cluster-4423.8589<br>Cluster-4423.14449<br>Cluster-4423.16998	K01937<br>K03023<br>K03014<br>K03022<br>K03022<br>K03783<br>K03019<br>K03002<br>K03006<br>K03020<br>K03009<br>K00254<br>K03021<br>K02999<br>K01493<br>K03004<br>K01956<br>K03002<br>K03014<br>K03005	*POLR2A RPC19, POLR1D RPB12, POLR2K DHODH, pyrD RPC2, POLR3B RPA1, POLR1A comEB RPA43 carA, CPA1 RPA2, POLR1B RPB6, POLR2F RPA49, POLR1E*
Steroid biosynthesis	27	107	0.00186179311278	0.0310298852129	Cluster-4423.12337<br>Cluster-4423.10876<br>Cluster-4423.12668<br>Cluster-4423.15963<br>Cluster-4423.19095<br>Cluster-4423.13991	K00559<br>K09831<br>K00637<br>K00222<br>K00559<br>K00559	*E2.1.1.41, SMT1, ERG6 ERG5 E2.3.1.26, SOAT TM7SF2, ERG24 E2.1.1.41, SMT1, ERG6 E2.1.1.41, SMT1, ERG6 E1.14.13.72, SC4MOL,*

					Cluster-4423.10815<br>Cluster-4423.18176<br>Cluster-4423.17896<br>Cluster-4423.18008<br>Cluster-4423.7362<br>Cluster-4423.13546<br>Cluster-4423.17548<br>Cluster-4423.13625<br>Cluster-4423.13774<br>Cluster-4423.13548<br>Cluster-4423.18598<br>Cluster-4423.13545<br>Cluster-4423.15835<br>Cluster-4423.13795<br>Cluster-4423.17103<br>Cluster-4423.15592<br>Cluster-4423.18449<br>Cluster-4423.15350<br>Cluster-4423.14849<br>Cluster-4423.14223<br>Cluster-4423.15490	K07750<br>K00637<br>K09831<br>K05917<br>K09831<br>K07750<br>K00511<br>K00227<br>K07750<br>K00637<br>K07750<br>K07750<br>K09831<br>K09829<br>K05917<br>K00801<br>K00559<br>K00801<br>K00559<br>K07750<br>K00637	*ERG25 E2.3.1.26,*<br>*SOAT ERG5 CYP51*<br>*ERG5 E1.14.13.72,*<br>*SC4MOL, ERG25*<br>*SQLE, ERG1 SC5DL,*<br>*ERG3 E1.14.13.72,*<br>*SC4MOL, ERG25*<br>*E2.3.1.26, SOAT*<br>*E1.14.13.72, SC4MOL,*<br>*ERG25 E1.14.13.72,*<br>*SC4MOL, ERG25*<br>*ERG5 ERG2 CYP51*<br>*FDFT1 E2.1.1.41,*<br>*SMT1, ERG6 FDFT1*<br>*E2.1.1.41, SMT1, ERG6*<br>*E1.14.13.72, SC4MOL,*<br>*ERG25 E2.3.1.26,*<br>*SOAT*

Table S3. The most enriched KEGG pathway terms down-regulated by ABA

Term	Sample number	Background number	P-value	Corrected P-value	UniGenes	KO	Gene name
Ubiquitin mediated proteolysis	42	169	1.19914824907e-05	0.000935010502169	Cluster-4423.8941<br>Cluster-4423.8042<br>Cluster-4423.11707<br>Cluster-4423.9725<br>Cluster-4423.14657<br>Cluster-4423.7056<br>Cluster-4423.18939<br>Cluster-4423.6470<br>Cluster-4423.4523<br>Cluster-4423.15292<br>Cluster-4423.13031<br>Cluster-4423.16698<br>Cluster-4423.6988<br>Cluster-4423.4325<br>Cluster-4423.17327<br>Cluster-4423.19532<br>Cluster-4423.20865<br>Cluster-4423.10490<br>Cluster-4423.19796<br>Cluster-4423.3454<br>Cluster-4423.20930	K10601<br>K06689<br>K10601<br>K03347<br>K03347<br>K10573<br>K10576<br>K03352<br>K10576<br>K03360<br>K10590<br>K10591<br>K03869<br>K03353<br>K03360<br>K03347<br>K10684<br>K10575<br>K10591<br>K03352<br>K03350	*SYVN1, HRD1*<br>*UBE2D_E, UBC4,*<br>*UBC5 SYVN1,*<br>*HRD1 CUL1,*<br>*CDC53 CUL1,*<br>*CDC53 UBE2A,*<br>*UBC2, RAD6A*<br>*UBE2H, UBC8*<br>*APC5 UBE2H,*<br>*UBC8 GRR1*<br>*TRIP12 NEDD4,*<br>*RSP5 CUL3 APC6,*<br>*CDC16 GRR1*<br>*CUL1, CDC53*<br>*UBLE1A, SAE1*<br>*UBE2G1, UBC7*<br>*NEDD4, RSP5*<br>*APC5 APC3,*<br>*CDC27 CDC4*<br>*CUL1, CDC53*<br>*UBE2A, UBC2,*

					Cluster-4423.18590<br>Cluster-4423.18988<br>Cluster-4423.8933<br>Cluster-4423.6469<br>Cluster-4423.5238<br>Cluster-4423.20698<br>Cluster-4423.17046<br>Cluster-4423.18104<br>Cluster-4423.8467<br>Cluster-4423.20001<br>Cluster-4423.3783<br>Cluster-4423.9990<br>Cluster-4423.17598<br>Cluster-4423.10509<br>Cluster-4423.5559<br>Cluster-4423.11402<br>Cluster-4423.15570<br>Cluster-4423.16970<br>Cluster-4423.16077<br>Cluster-4423.13077<br>Cluster-4423.17377	K03361<br>K03347<br>K10573<br>K03869<br>K10685<br>K03347<br>K02207<br>K03360<br>K10573<br>K03347<br>K10684<br>K03361<br>K10589<br>K03361<br>K10579<br>K10601<br>K02207<br>K10259<br>K10575<br>K10575<br>K03360	*RAD6A CUL3*<br>*UBLE1B, SAE2,*<br>*UBA2 CUL1,*<br>*CDC53 UBE2R,*<br>*UBC3, CDC34*<br>*GRR1 UBE2A,*<br>*UBC2, RAD6A*<br>*CUL1, CDC53*<br>*UBLE1A, SAE1*<br>*CDC4 UBE3C*<br>*CDC4 UBE2M,*<br>*UBC12 SYVN1,*<br>*HRD1 UBE2R,*<br>*UBC3, CDC34*<br>*MET30 UBE2G1,*<br>*UBC7 UBE2G1,*<br>*UBC7 GRR1*
Cell cycle - yeast	57	268	2.08927742368e-05	0.000935010502169	Cluster-4423.10352<br>Cluster-4423.4945<br>Cluster-4423.4946<br>Cluster-4423.23194<br>Cluster-4423.22331	K03456<br>K06641<br>K06641<br>K06636<br>K02607	*PPP2R1 CHK2*<br>*CHK2 SMC1 ORC5*<br>*CUL1, CDC53*<br>*CUL1, CDC53*<br>*SMC1 SWE1 TUP1*

					Cluster-4423.9725<br>Cluster-4423.14657<br>Cluster-4423.22790<br>Cluster-4423.4686<br>Cluster-4423.19666<br>Cluster-4423.17015<br>Cluster-4423.6470<br>Cluster-4423.18422<br>Cluster-4423.4325<br>Cluster-4423.15292<br>Cluster-4423.3109<br>Cluster-4423.18755<br>Cluster-4423.17612<br>Cluster-4423.20001<br>Cluster-4423.13419<br>Cluster-4423.10576<br>Cluster-4423.20698<br>Cluster-4423.10574<br>Cluster-4423.17327<br>Cluster-4423.16985<br>Cluster-4423.16702<br>Cluster-4423.13082<br>Cluster-4423.19532<br>Cluster-4423.3454<br>Cluster-4423.20930<br>Cluster-4423.18590	K03347<br>K03347<br>K06636<br>K03114<br>K06666<br>K02214<br>K03352<br>K06653<br>K03353<br>K03360<br>K06674<br>K02179<br>K04382<br>K03347<br>K06653<br>K06677<br>K03347<br>K06677<br>K03360<br>K02542<br>K02540<br>K03114<br>K03347<br>K03352<br>K03350<br>K03361	*CDC7 APC5*<br>*PHO81 APC6,*<br>*CDC16 GRR1*<br>*SMC2 BUB2*<br>*PPP2C CUL1,*<br>*CDC53 PHO81*<br>*YCS4, CNAP1,*<br>*CAPD2 CUL1,*<br>*CDC53 YCS4,*<br>*CNAP1, CAPD2*<br>*GRR1 MCM6*<br>*MCM2 SWE1*<br>*CUL1, CDC53*<br>*APC5 APC3,*<br>*CDC27 CDC4*<br>*SMC1 SMC1 CDC4*<br>*HRAD1, RAD17*<br>*PPP2R1 MET30*<br>*GRR1 BUB2*<br>*PPP2C MCM7,*<br>*CDC47 MCM7,*<br>*CDC47 BUB2*<br>*SCC2, NIPBL*<br>*CHK2 YCS4,*<br>*CNAP1, CAPD2*<br>*YCS4, CNAP1,*

					Cluster-4423.3315<br>Cluster-4423.21679<br>Cluster-4423.9990<br>Cluster-4423.5822<br>Cluster-4423.18063<br>Cluster-4423.16970<br>Cluster-4423.18104<br>Cluster-4423.17100<br>Cluster-4423.8354<br>Cluster-4423.23329<br>Cluster-4423.2640<br>Cluster-4423.15675<br>Cluster-4423.11191<br>Cluster-4423.11064<br>Cluster-4423.21952<br>Cluster-4423.10401<br>Cluster-4423.10509<br>Cluster-4423.15961<br>Cluster-4423.13148<br>Cluster-4423.12262<br>Cluster-4423.21339<br>Cluster-4423.18988<br>Cluster-4423.9330<br>Cluster-4423.16965<br>Cluster-4423.21593<br>Cluster-4423.17377	K06636<br>K06636<br>K03361<br>K02830<br>K03456<br>K10259<br>K03360<br>K02179<br>K04382<br>K02210<br>K02210<br>K02179<br>K06672<br>K06641<br>K06677<br>K06677<br>K03361<br>K02180<br>K06641<br>K03456<br>K02210<br>K03347<br>K06677<br>K02542<br>K06636<br>K03360	*CAPD2 CDC4 BUB3 CHK2 PPP2R1 MCM7, CDC47 CUL1, CDC53 YCS4, CNAP1, CAPD2 MCM6 SMC1 GRR1*

Basal transcription factors	20	56	4.42503982727e-05	0.000935010502169	Cluster-4423.7909 Cluster-4423.8211 Cluster-4423.5347 Cluster-4423.13597 Cluster-4423.3903 Cluster-4423.11446 Cluster-4423.5072 Cluster-4423.13341 Cluster-4423.10746 Cluster-4423.9220 Cluster-4423.21967 Cluster-4423.15962 Cluster-4423.2706 Cluster-4423.8859 Cluster-4423.5887 Cluster-4423.3906 Cluster-4423.3320 Cluster-4423.5888 Cluster-4423.9441 Cluster-4423.5702	K03132 K10842 K03139 K03126 K03123 K10843 K03123 K03125 K03139 K03131 K03142 K03120 K03136 K03125 K10844 K03139 K03136 K10844 K03122 K03142	*TAF7 MNAT1 TFIIF2, GTF2F2, TFG2 TAF12 TFIIA2, GTF2A2, TOA2 ERCC3, XPB TFIIA2, GTF2A2, TOA2 TAF1 TFIIF2, GTF2F2, TFG2 TAF6 TFIIH2, GTF2H2, SSL1 TBP, tbp TFIIE1, GTF2E1, TFA1, tfe TAF1 ERCC2, XPD TFIIF2, GTF2F2, TFG2 TFIIE1, GTF2E1, TFA1, tfe ERCC2, XPD TFIIA1, GTF2A1, TOA1 TFIIH2, GTF2H2, SSL1*
Protein processing in endoplasmic reticulum	50	231	4.51555507918e-05	0.000935010502169	Cluster-4423.4197 Cluster-4423.8941 Cluster-4423.11707 Cluster-4423.8042 Cluster-4423.9238	K14007 K10601 K10601 K06689 K14026	*SEC24 SYVN1, HRD1 SYVN1, HRD1 UBE2D_E, UBC4, UBC5 SEL1, SEL1L WBP1*

					Cluster-4423.3343<br>Cluster-4423.2868<br>Cluster-4423.9725<br>Cluster-4423.14657<br>Cluster-4423.8903<br>Cluster-4423.20038<br>Cluster-4423.15367<br>Cluster-4423.8056<br>Cluster-4423.15678<br>Cluster-4423.11402<br>Cluster-4423.19363<br>Cluster-4423.9383<br>Cluster-4423.5157<br>Cluster-4423.18240<br>Cluster-4423.5527<br>Cluster-4423.11241<br>Cluster-4423.10240<br>Cluster-4423.8905<br>Cluster-4423.12562<br>Cluster-4423.14694<br>Cluster-4423.19532<br>Cluster-4423.10490<br>Cluster-4423.15548<br>Cluster-4423.16077<br>Cluster-4423.10860<br>Cluster-4423.19815	K12670<br>K12667<br>K03347<br>K03347<br>K14018<br>K09523<br>K09490<br>K10956<br>K09490<br>K10601<br>K09518<br>K14018<br>K10956<br>K01230<br>K07151<br>K14006<br>K08860<br>K05546<br>K14022<br>K01230<br>K03347<br>K10575<br>K08852<br>K10575<br>K10661<br>K13996	*SWP1, RPN2 CUL1,*<br>*CDC53 CUL1,*<br>*CDC53 PLAA,*<br>*DOA1, UFD3*<br>*DNAJC3 HSPA5,*<br>*BIP SEC61A*<br>*HSPA5, BIP*<br>*SYVN1, HRD1*<br>*DNAJB12 PLAA,*<br>*DOA1, UFD3*<br>*SEC61A MAN1*<br>*STT3 SEC23*<br>*EIF2AK3 GANAB*<br>*CUE1, KIS4 MAN1*<br>*CUL1, CDC53*<br>*UBE2G1, UBC7*<br>*ERN1 UBE2G1,*<br>*UBC7 MARCH6,*<br>*DOA10 EPS1*<br>*SHP1, UBX1,*<br>*NSFL1C CUL1,*<br>*CDC53 HSPA1_8*<br>*MAN1 SEC23*<br>*MAN1 CUL1,*<br>*CDC53 HYOU1*<br>*MAN1 NPLOC4,*

					Cluster-4423.6803<br>Cluster-4423.18988<br>Cluster-4423.16594<br>Cluster-4423.14696<br>Cluster-4423.8232<br>Cluster-4423.15199<br>Cluster-4423.20001<br>Cluster-4423.8542<br>Cluster-4423.9016<br>Cluster-4423.6770<br>Cluster-4423.6772<br>Cluster-4423.21348<br>Cluster-4423.6416<br>Cluster-4423.23683<br>Cluster-4423.18565<br>Cluster-4423.18729<br>Cluster-4423.10254<br>Cluster-4423.20698<br>Cluster-4423.13077	K14012<br>K03347<br>K03283<br>K01230<br>K14006<br>K01230<br>K03347<br>K09486<br>K01230<br>K14015<br>K14015<br>K10956<br>K14026<br>K03283<br>K01230<br>K01230<br>K01228<br>K03347<br>K10575	*NPL4 NPLOC4,*<br>*NPL4 SEC61A*<br>*SEL1, SEL1L*<br>*HSPA1_8 MAN1*<br>*MAN1 GCS1 CUL1,*<br>*CDC53 UBE2G1,*<br>*UBC7*
Proteasome	31	115	4.53888593286e-05	0.000935010502169	Cluster-4423.4982<br>Cluster-4423.4883<br>Cluster-4423.7980<br>Cluster-4423.21427<br>Cluster-4423.6619<br>Cluster-4423.7054<br>Cluster-4423.6324	K02738<br>K02735<br>K03062<br>K02726<br>K02736<br>K03036<br>K02725	*PSMB6 PSMB3*<br>*PSMC1, RPT2*<br>*PSMA2 PSMB4*<br>*PSMD11, RPN6*<br>*PSMA1 PSMD1,*<br>*RPN2 PSMC4,*<br>*RPT3 PSMB6*

					Cluster-4423.7215<br>Cluster-4423.12923<br>Cluster-4423.5336<br>Cluster-4423.20385<br>Cluster-4423.2492<br>Cluster-4423.9022<br>Cluster-4423.7979<br>Cluster-4423.6071<br>Cluster-4423.11008<br>Cluster-4423.4597<br>Cluster-4423.13676<br>Cluster-4423.6681<br>Cluster-4423.11063<br>Cluster-4423.22250<br>Cluster-4423.11315<br>Cluster-4423.20296<br>Cluster-4423.4500<br>Cluster-4423.5753<br>Cluster-4423.11053<br>Cluster-4423.7396<br>Cluster-4423.16004<br>Cluster-4423.6162<br>Cluster-4423.6386<br>Cluster-4423.4488	K03032<br>K03063<br>K02738<br>K02727<br>K03029<br>K02727<br>K03062<br>K03035<br>K03038<br>K02738<br>K03065<br>K03028<br>K03064<br>K02734<br>K03031<br>K02729<br>K03029<br>K03032<br>K11599<br>K02739<br>K03061<br>K02738<br>K02738<br>K02731	*PSMA3 PSMD4, RPN10 PSMA3 PSMC1, RPT2 PSMD12, RPN5 PSMD7, RPN8 PSMB6 PSMC3, RPT5 PSMD2, RPN1 PSMC6, RPT4 PSMB2 PSMD8, RPN12 PSMA5 PSMD4, RPN10 PSMD1, RPN2 POMP, UMP1 PSMB7 PSMC2, RPT1 PSMB6 PSMB6 PSMA7*
